# Supplementary material for: Front-of-pack nutritional labels: Understanding by low- and middle-income Mexican consumers
Source: PLoS One. 2019 Nov 18;14(11):e0225268. doi: 10.1371/journal.pone.0225268 (PMC6860442; doi:10.1371/journal.pone.0225268)
Supplement: S1 Fig — (DOCX) [file pone.0225268.s001.docx]

**EXAMPLES OF DOUBLE-SIZED BOARDS**


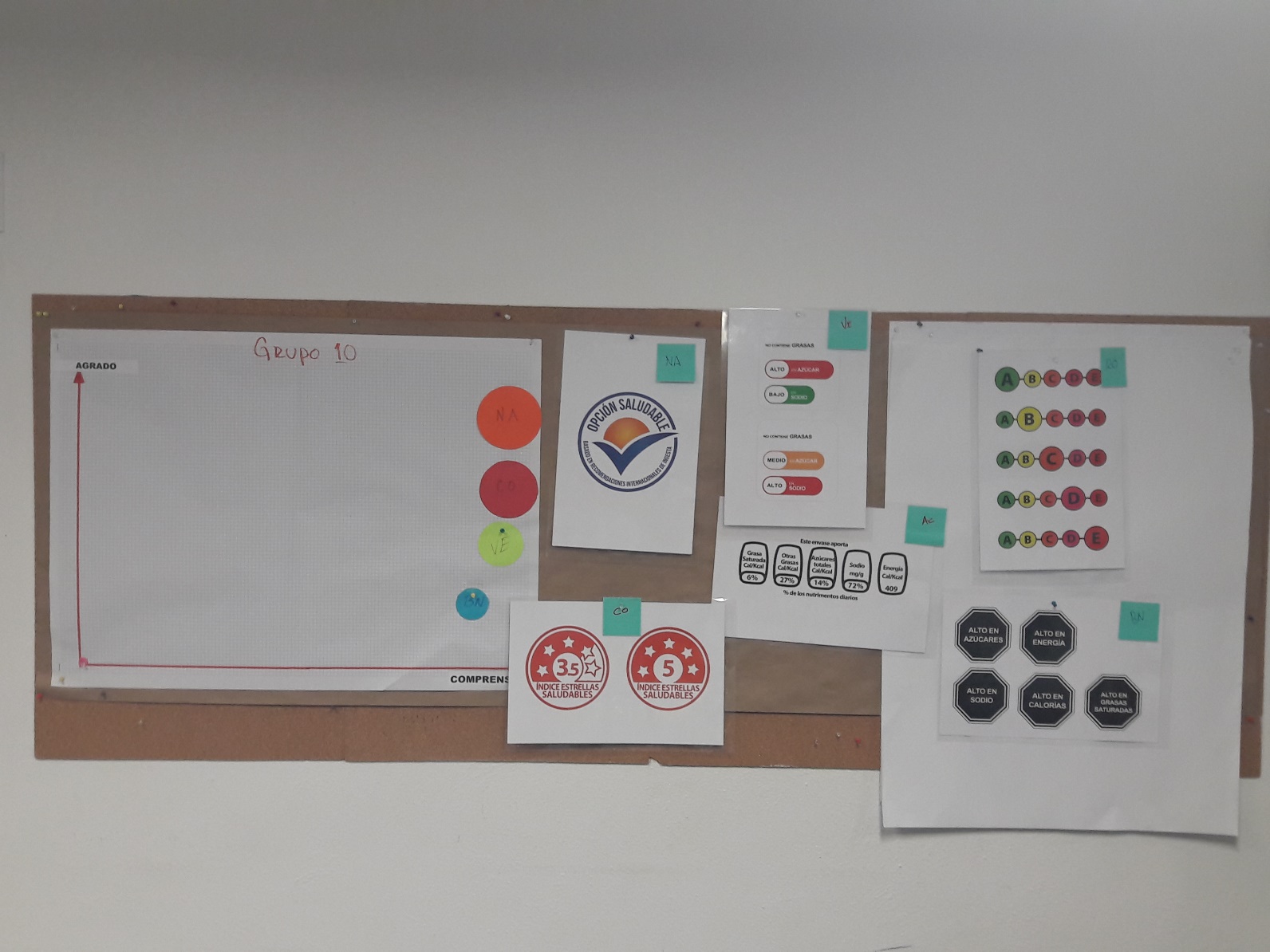

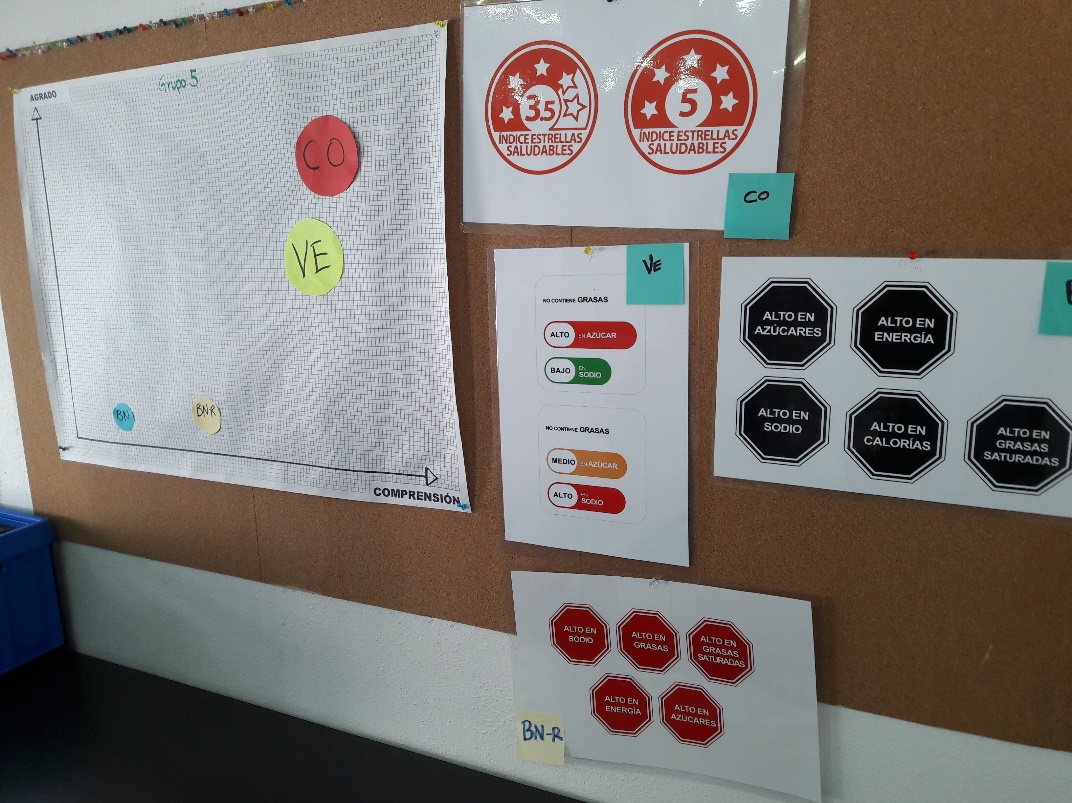


**
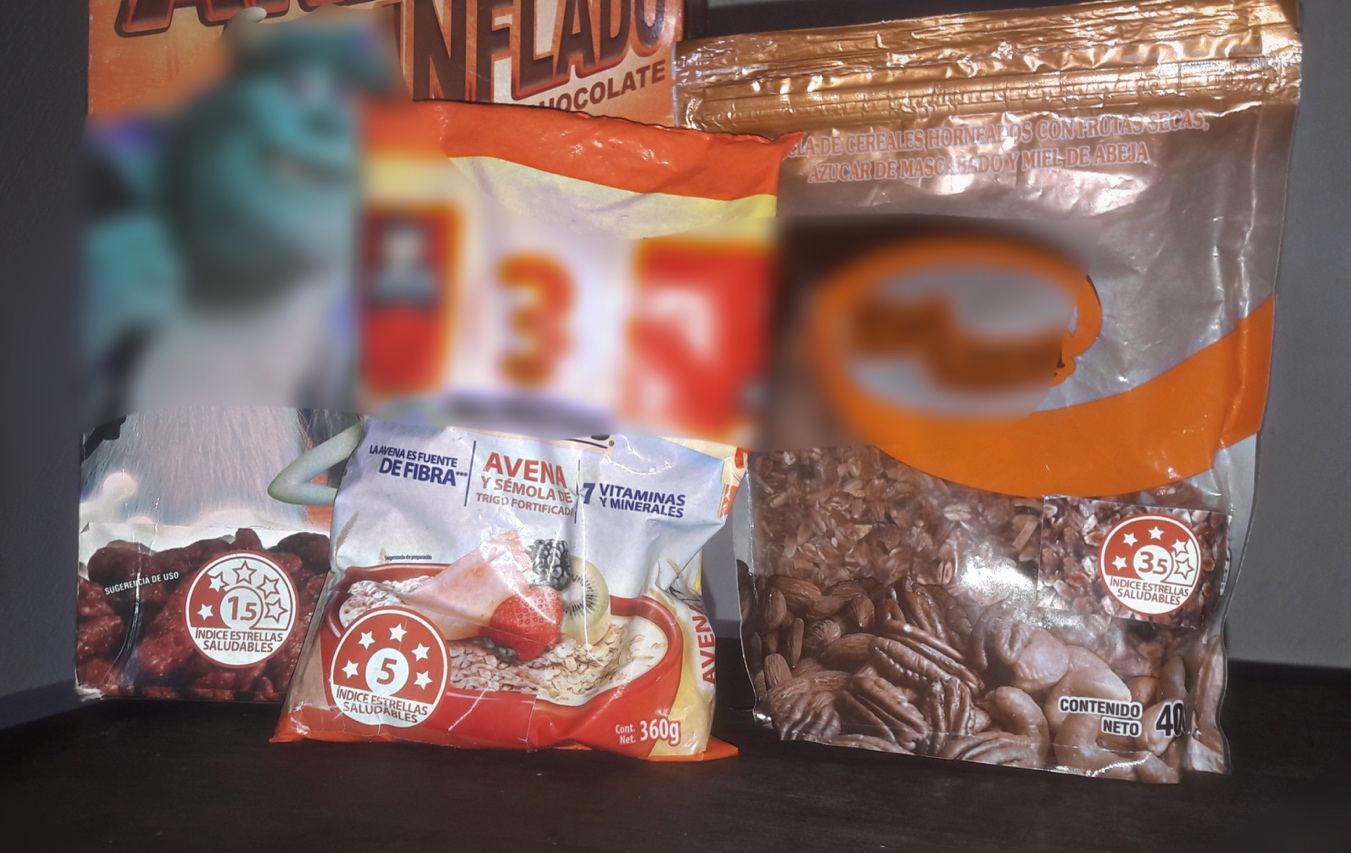
EXAMPLES OF PRODU
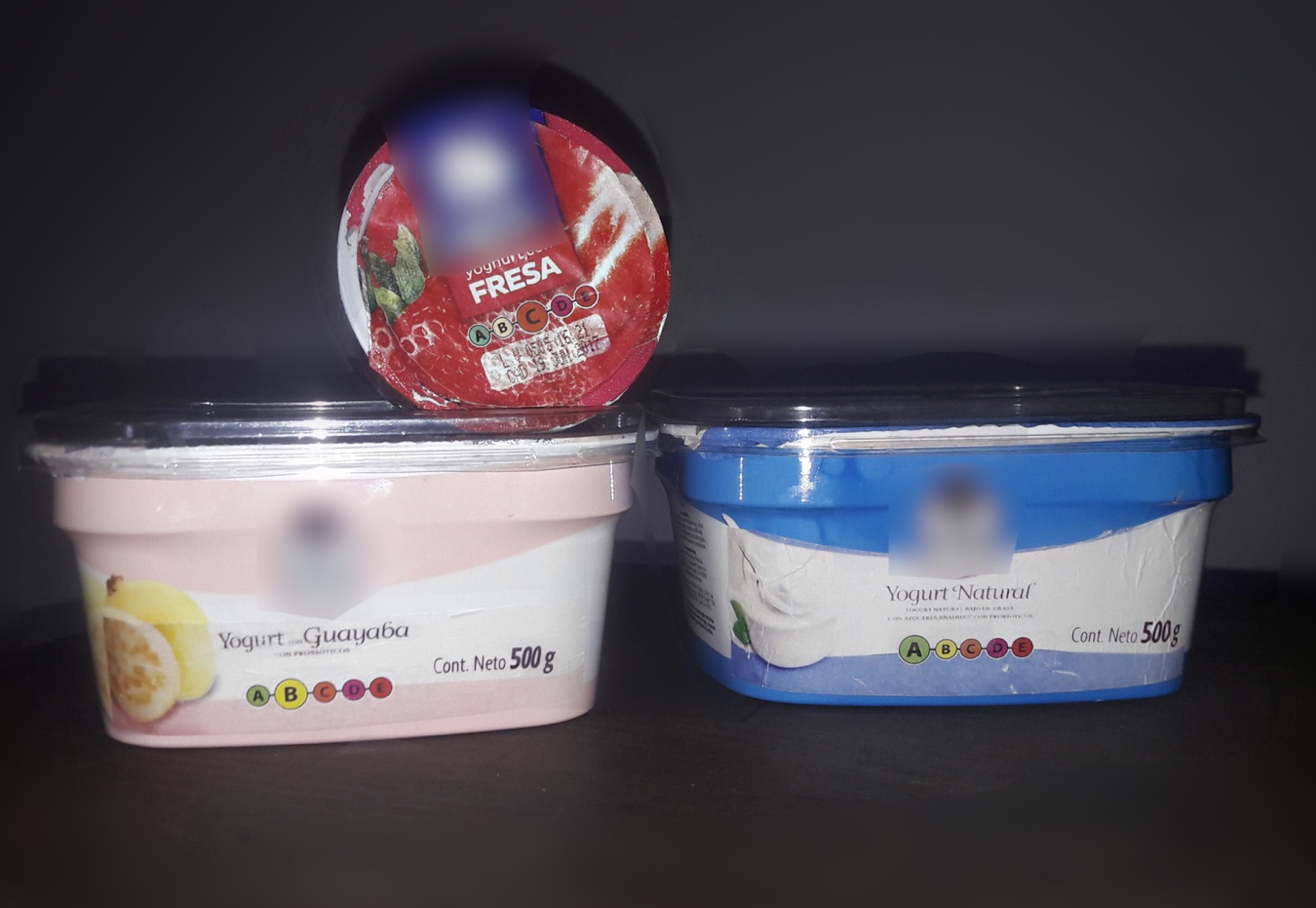
CTS CATEGORIES WITHIN THE SAME LABEL**

**
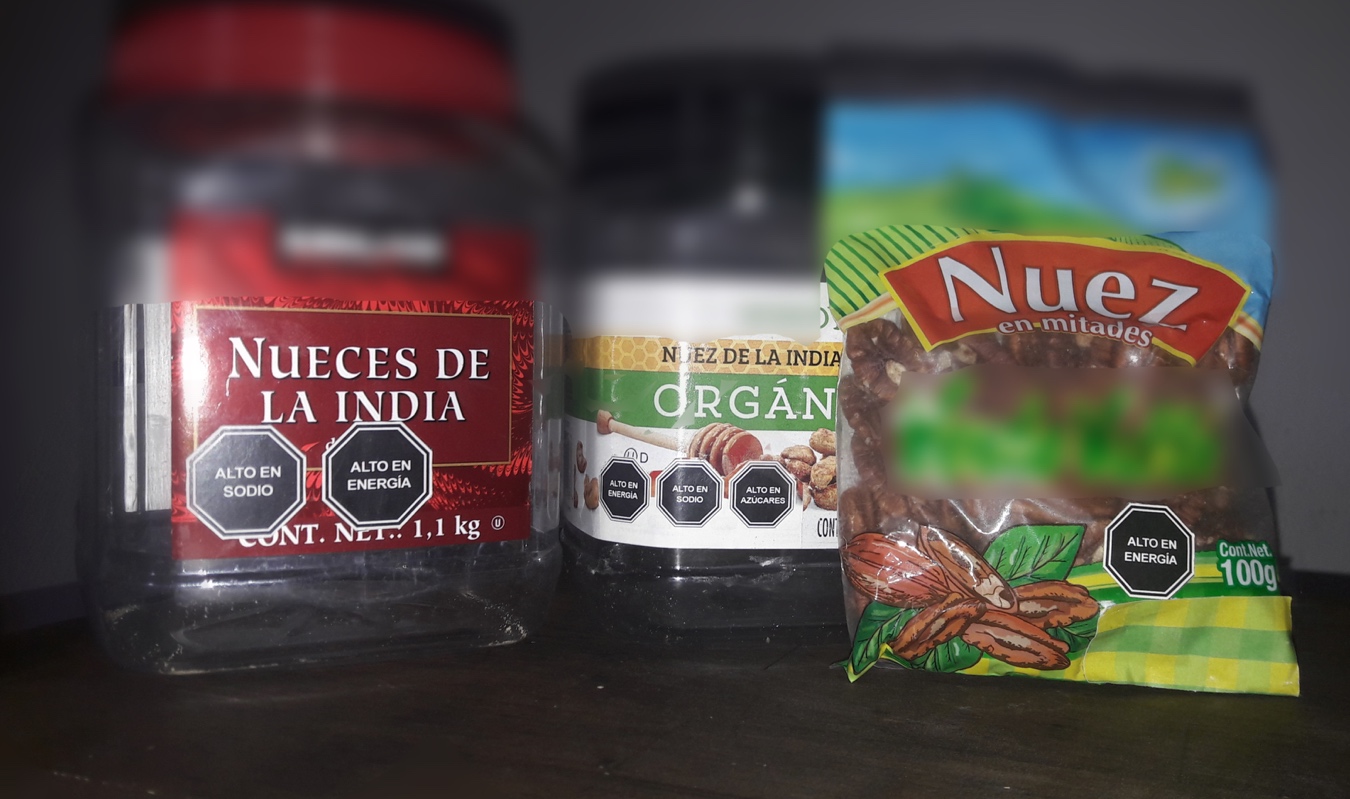
**

**
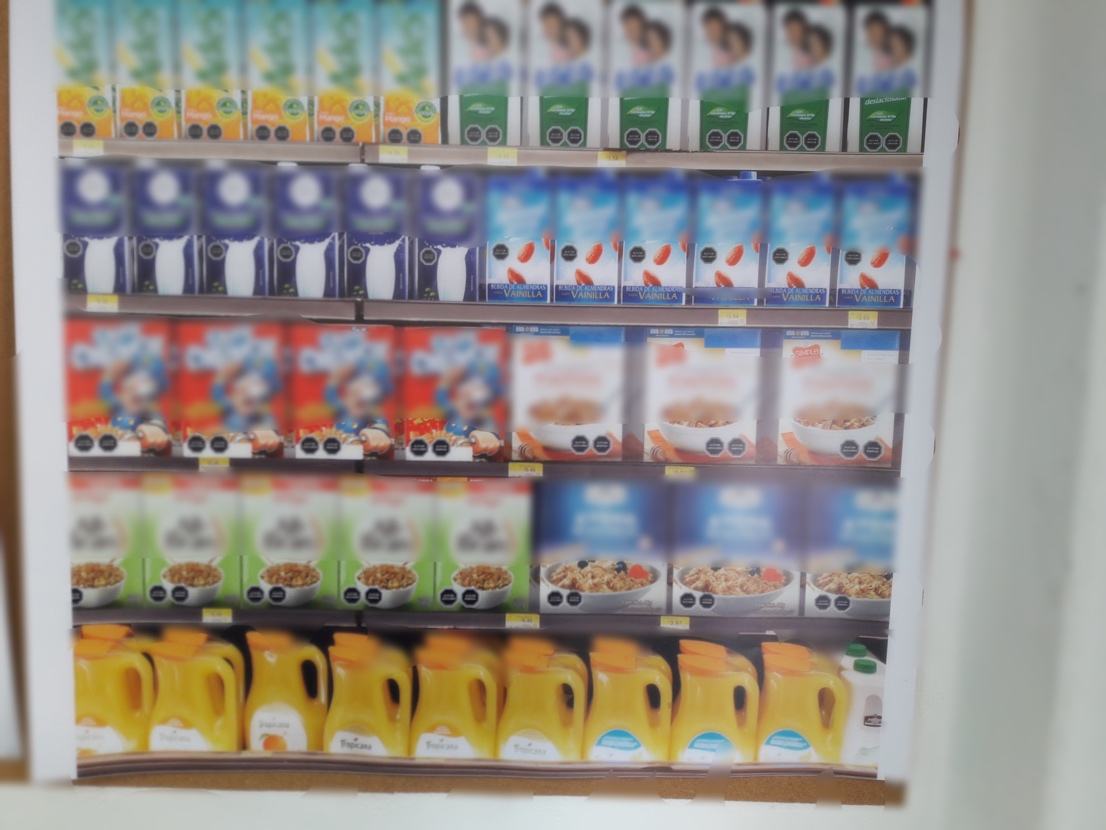
EXAMPLES OF POSTERS**

**
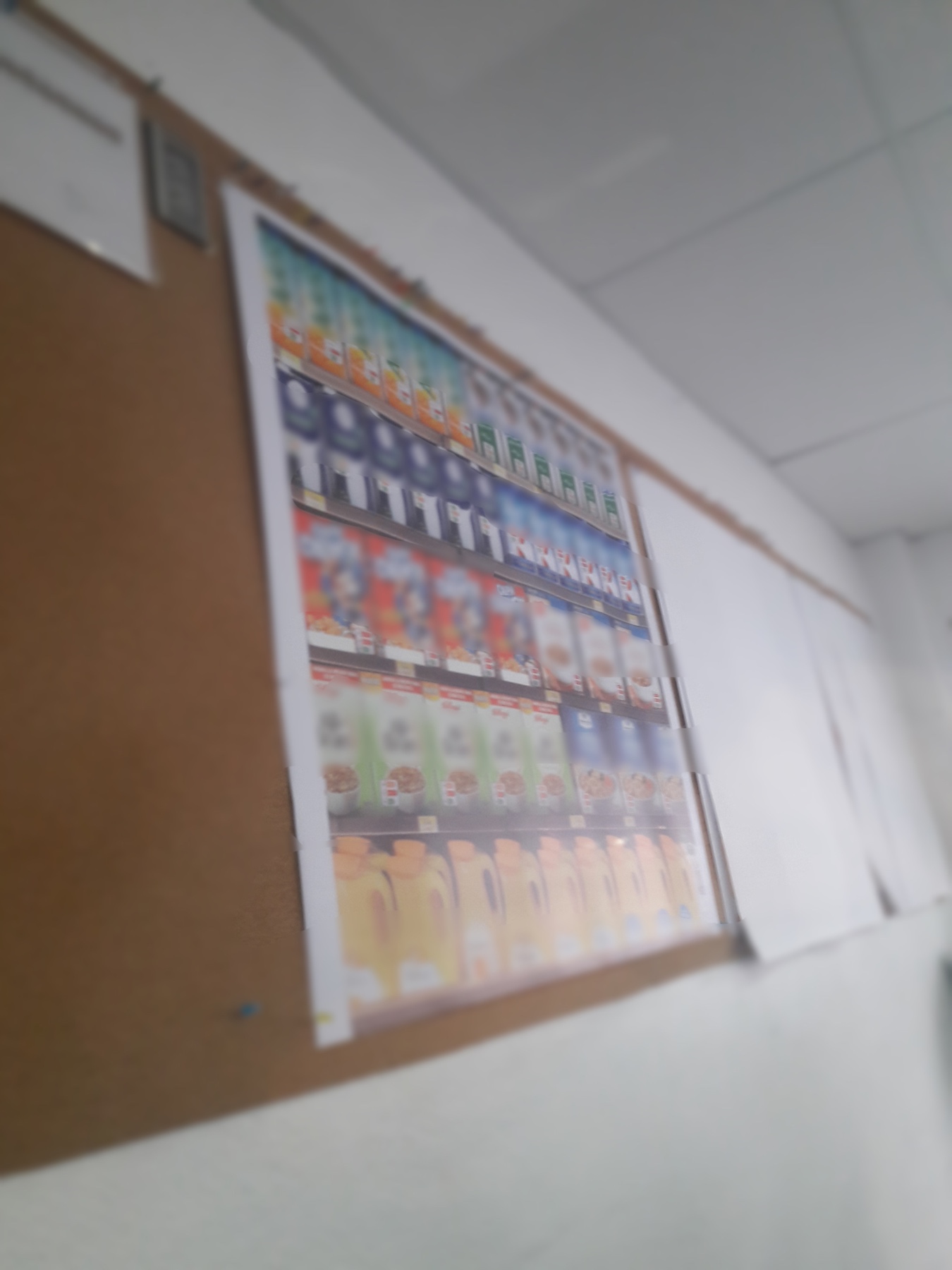
**
