# Supplementary material for: Front-of-pack nutritional labels: Understanding by low- and middle-income Mexican consumers
Source: PLoS One. 2019 Nov 18;14(11):e0225268. doi: 10.1371/journal.pone.0225268 (PMC6860442; doi:10.1371/journal.pone.0225268)
Supplement: S1 Table — SES, socio economic status; M, male; F, female. (DOCX) [file pone.0225268.s002.docx]

| **S1 Table. Characteristics of the participating groups/ sessions.** | | | | | | |
| --- | --- | --- | --- | --- | --- | --- |
| **Groups** | **n** | **SES** | | **Group/ profile** | **Age**  Mean (range) | **Gender** |
|  |  | **Medium** | **Low** |  |  |  |
| 4 | 40 | 20 | 20 | Adolescents | 14.1 (13-15) | 23 M – 17 F |
| 4 | 40 | 20 | 20 | Young Adults | 21.9 (21-23) | 21 M – 19 F |
| 4 | 40 | 20 | 20 | Mothers with children between 3 and 12 years | 36.9 (23-50) | 40 F |
| 4 | 40 | 20 | 20 | Fathers with children between 3 and 12 years | 38.5 (26-54) | 40 M |
| 4 | 40 | 20 | 20 | Older Adults | 60.6 (55-70) | 20 M- 20 F |
| SES, socio economic status; M, male; F, female. | | | | | | |
