# Supplementary material for: Front-of-pack nutritional labels: Understanding by low- and middle-income Mexican consumers
Source: PLoS One. 2019 Nov 18;14(11):e0225268. doi: 10.1371/journal.pone.0225268 (PMC6860442; doi:10.1371/journal.pone.0225268)
Supplement: S2 Table — 5-CNL, 5 Color Nutritional Labelling; MTL, Multiple Traffic Light; GDA, Guide Dairy Amounts; HSR, Health Ranting Stars. (DOCX) [file pone.0225268.s003.docx]

| **S2 Table. Nutritional content and classification of products utilized.** | | | | | | | | |
| --- | --- | --- | --- | --- | --- | --- | --- | --- |
| **Product** | **FOPL** | **Nutritional content**  **Per 100 g/ml** | | | | | | **Nutrition Quality** |
|  |  | **Energy**  **Kcal** | **Sodium/**  **salt**  **mg** | **Total sugar**  **g** | **Saturated Fat**  **g** | **Trans Fat**  **g** | **Added sugar**  **g** |  |
| Natural walnuts | Warning black/red | 639 | 2 | 3 | 3.4 | 0 | 0 | High |
| Cashews | Warning black/red | 563.3 | 400 | 26.6 | 8.3 | 0 | 0 | Medium |
| Cashews with honey | Warning black/red | 526 | 226.6 | 44.3 | 5.7 | 0 | 28.3 | Low |
| Natural yogurt | 5- CNL | 57.7 | 52 | 6.3 | 1.1 | 0 | 6.3 | High |
| Guava yogurt | 5- CNL | 82.9 | 39.5 | 14.1 | 1 | 0 | 12.3 | Medium |
| Strawberry yogurt | 5- CNL | 99.3 | 48.3 | 15.4 | 1.9 | 0 | 14.7 | Low |
| Orange flavor soy juice | MTL | 21 | 11.5 | 3.8 | 0.1 | 0 | 3.7 | High |
| Natural orange juice | MTL | 217.6 | 3.2 | 12.4 | 0 | 0 | 12.4 | Medium |
| Mango flavor drink | MTL | 45 | 4.2 | 11.3 | 0 | 0 | 10.8 | Low |
| Natural oats | HSR | 343.3 | 0 | 61 | 1 | 0 | 2 | High |
| Granola with honey | HSR | 440 | 0 | 70 | 2.5 | 0 | 22 | Medium |
| Puffed rice cereal | HSR | 188.5 | 236.6 | 43.6 | 0.3 | 0 | 16.5 | Low |
| Black bean burger | GDA | 182.4 | 504 | 25.4 | .8 | 0 | 2.8 | Low |
| Milk | GDA | 40.8 | 50 | 4.8 | 0.7 | 0 | 0 | High |
| Sesame bar | GDA | 226.5 | 0 | 25.8 | 0 | 0 | 0 | Medium |
| Natural almonds | Healthy Choices | 546 | 1 | 4 | 3.7 | 0 | 0 | High |
| Natural orange juice | Healthy Choices | 217.6 | 3.2 | 12.4 | 0 | 0 | 12.4 | High |
| Natural walnuts | Healthy Choices | 639 | 2 | 3 | 3.4 | 0 | 0 | Low |
| *5-CNL, 5 Color Nutritional Labelling; MTL, Multiple Traffic Light; GDA, Guide Dairy Amounts; HSR, Health Ranting Stars.* | | | | | | | | |
