# Supplementary material for: Front-of-pack nutritional labels: Understanding by low- and middle-income Mexican consumers
Source: PLoS One. 2019 Nov 18;14(11):e0225268. doi: 10.1371/journal.pone.0225268 (PMC6860442; doi:10.1371/journal.pone.0225268)
Supplement: S1 File — (DOC) [file pone.0225268.s004.doc]

**Etiquetado frontal nutrimental de alimentos industrializados para consumidores mexicanos**

**Introducción**

Hola, mi nombre es ______________, estamos efectuando el estudio ahora sino solo entrevistando a posibles candidatos que deseen formar parte de esa investigación que será efectuada en el mes de marzo de 2017.

Todas las personas que formen parte de esta investigación recibirán $ 400 en monedero electrónico como una forma de agradecimiento por su tiempo. ¿Me permitiría hacerle unas cuantas preguntas con la finalidad de saber si es usted elegible para esta investigación?

1. Registrar género:

- Hombre (checar cuotas de reclutamiento)
- Mujer (checar cuotas de reclutamiento)

1. ¿Me podría indicar su edad? *[Permitir sólo una respuesta]*

- 13 a 15 años de edad *[Continuar]*
- De 16 a 20 años de edad *[Terminar]*
- De 21 a 23 años de edad *[Pasar a P5]*
- Mayor de 24 años *[Terminar]*

1. ¿En este momento lo acompaña alguno de sus padres o tutor que nos permita hacerle la entrevista?

- Sí (pedir autorización y continuar)

Nombre y firma del padre ___________________

- No (Terminar)

1. De ser elegible de acuerdo a sus respuestas ¿Considera que sus padres o tutores estarían dispuestos a acompañarlo a las instalaciones donde se llevará a cabo dicha investigación y esperarlo en recepción mientras participa usted en ella?

- Sí (continuar, y al final firmar autorización por parte del padre o tutor)
- No (Terminar)

1. ¿Trabaja usted o alguno de los familiares que integran su núcleo familiar en alguna de las siguientes industrias?

- Promoción de la Salud (agradecer y **terminar**)
- Investigación de Mercados (agradecer y **terminar**)
- Publicidad (agradecer y **terminar**)
- Industria de Bebidas (agradecer y **terminar**)
- Alimentos empacados (agradecer y **terminar**)
- Ninguna de las anteriores (continuar)

1. ¿Sabe usted leer y escribir en español?

- Sí (continuar)
- No (agradecer y terminar)

1. Hablando de la compra de alimentos, botana y en general alimentos pre empacados, me podría indicar ¿Cuál es la frecuencia con la que compra y consume este tipo de productos? *[Permitir sólo una respuesta]*

- Mensualmente [Terminar]
- Quincenalmente [Terminar]
- Semanalmentec [Terminar]
- Más de una vez por semana (continuar)

1. ¿Qué tipo de alimentos, botana y en general alimentos pre empacados son los que acostumbra comprar consumir? *[multirespuesta]*

- Cereales empacados (Galletas, pastelitos, cereal de caja, harina de hot cakes, etc.)
- Bebidas envasadas (jugos, refrescos, te´s, etc.)
- Botanas saladas (cacahuates, papas, nueces, etc.)
- Alimentos pre preparados o listos para comer (sandwiches, tamales, pizzas, nuggets, etc.)
- Lacteos (leche, yogurt, queso, etc.)
- Ninguno de los anteriores [Terminar]

Si contesta al menos dos de los productos mencionados en la lista será seleccionado a participar.

Con base en el tipo de respuestas que usted nos proporcionó ha resultado elegible para participar en el estudio de investigación que le habíamos mencionado.

Quienes participen en esta investigación formarán parte de un grupo de aproximadamente de 10 personas quienes platicarán respecto a temas relacionados con alimentos empacados y bebidas envasadas. La reunión tendrá una duración aproximada de 105 minutos (1:45 horas) a partir de que inicie el grupo.

Toda la información que usted nos proporcione será manejada en forma confidencial y usted recibirá un monedero electrónico con $ 400 pesos MN en agradecimiento por su tiempo

1. ¿Está usted interesado en participar en este estudio?

- Sí (continuar)
- No (agradecer y **terminar**)

El grupo para el que usted fue elegido se realizará el día __ (fecha) a las ________ (hora). En la siguiente dirección __________________________________

_________________________________________________________________

1. ¿Está usted disponible para participar en este día y horario?

- Sí (continuar).
- No (agradecer y **terminar**)

Agradecer y rectificar fecha, horario y lugar.

Recuérdele la existencia del incentivo y asegúrese de proporcionarle sus datos para que pueda reportarse y cancelar si es que se le complica asistir a la sesión o viene retrasado a su cita!

**Front nutrition labeling of industrialized foods for Mexican consumers**

**Introduction**

Hello, my name is ______________, and stamos conducting the study but only now interviewing potential candidates who wish to be part of that research will be carried out in March 2017 .

All persons forming p a rte of this research will receive $ 400 in electronic wallet as a way of thanks for your time. Would you allow me to ask you a few questions in order to know if you are eligible for this research?

1. Register gender:

🞎Homb r e (check recruitment quotas)

🞎Woman (check recruitment fees)

1. Could you tell me your age? *[Allow only one answer]*

🞎13 to 15 years old *[Continue]*

🞎16 to 20 years old *[Finish]*

🞎From 21 to 23 years old *[Go to P5]*

🞎Mayor 24 to ñ os *[Finish]*

1. Are you currently accompanied by a parent or guardian who allows us to do the interview?

🞎Yes ( ask for authorization and continue)

Name and signature of the father ___________________

🞎No ( Finish )

1. To be eligible according to their res p uestas ¿ Considers that s US parents or guardians would be willing to accompany him to venues will be out this investigation ON and wait in reception ON while you participate in it?

🞎Yes (continue , and eventually signed authorization part and the parent or guardian )

🞎No (Finish)

1. Do you or any of the family members who integrate your family in any of the following industries work?

🞎Health Promotion (thank and **finish**)

🞎Market Research       (Thank e r and **finish**)

🞎Advertising (thank and **finish**)

🞎Beverage Industry    (thank and **finish**)

🞎Packaged food              (thank and **finish**)

🞎None of the above (continue)

1. ¿ You know read and escrib i r in Spanish?

🞎Yes (continue)

🞎No (thank and finish)

1. Talk about the purchase of food , snacks and in general pre-packaged food , could you tell me what is the frequency with which you buy and consume this type of products ? *[Allow only one respue s ta]*

🞎Monthly [Finish]

🞎Biweekly [Finish]

🞎Weeklyc [Finish]

🞎More than once a week (continue)

1. What kind of foods, snacks and general pre-packaged foods are the ones you usually buy? *[multi-response]*

🞎Packaged cereals (Cookies, cupcakes, box cereal, hot cakes flour, etc.)

🞎Packaged drinks (juices, soft drinks, tea, etc.)

🞎Salted snacks (peanuts, potatoes, nuts, etc.)

🞎Pre-prepared or ready-to-eat foods (sandwiches, tamales , pizzas, nugge t s, etc.)

🞎Dairy products (milk, yogurt, cheese, etc.)

🞎None of the above [End]

If you answer at least two of the products mentioned in the list you will be selected to participate.

Based on the type of responses you provided to us, you have been eligible to participate in the research study that we mentioned.

Those participating in this research will be part of a group of approximately of 10 people who platicarán on issues related to food packs d os and bottled drinks s. The meeting will last approximately 105 minutes (1: 4 5 hours) from the start of the group .

All the information you provide to us will be handled confidentially and you will receive an electronic wallet with n $ 400 pesos in appreciation for your time.

1. Are you interested in participating in this study?

🞎Yes (continue)

🞎No (thank and **finish**)

The group for which you were chosen will be held on __   (date) at ________ (time). In the NEXT n will address __________________________________

_________________________________________________________________

1. Are you available to participate in this day and time?

🞎Yes (continue).

🞎No (thank and **finish**)

Acknowledge and correct date, h orario and place.

Remind him of the existence of the incentive and be sure to provide him with his information so that he can report and cancel if he is complicated to attend the session or is late for his appointment!
