# Supplementary material for: Front-of-pack nutritional labels: Understanding by low- and middle-income Mexican consumers
Source: PLoS One. 2019 Nov 18;14(11):e0225268. doi: 10.1371/journal.pone.0225268 (PMC6860442; doi:10.1371/journal.pone.0225268)
Supplement: S2 File — (DOCX) [file pone.0225268.s005.docx]

**EVALUACIÓN DE LA COMPRENSIÓN Y ACEPTABILIDAD DE SISTEMAS DE ETIQUETADO FRONTAL**

**Guía de tópicos para el moderador**

**Propósito Central**

***Recordemos que el propósito central de este estudio es ayudar a los consumidores, a través del etiquetado frontal en productos alimenticios pre-empacados, a hacer elecciones de comida más saludable***

**Tópicos**

La siguiente guía de tópicos provee una visión en general de las áreas que se cubrirán a través de la exploración y prueba de las diversas alternativas de etiquetado frontal. No se pretende que está guía sea una lista de preguntas. La sesión puede fluir de manera libre, el moderador buscará captar las reacciones, pensamientos y el discurso espontáneo como vaya surgiendo, garantizando que todas las partes claves fueron cubiertas

Un promedio de 3 a 5 alternativas de etiquetado frontal se examinarán durante los grupos de enfoque, se mostrarán monádicamente, de manera aleatoria y procurando un orden distinto en cada grupo, para dar opción a que la mayoría de ellos tengan la oportunidad de aparecer en primera posición.

El flujo de los grupos se puede resumir de la siguiente manera:

**1. Introducción 5 min**

**2. Elección de alimentos pre-empacados 15 min**

**30 minutes**

**3. Evaluación aceptabilidad y comprensión de Etiquetados 90 min**

**12 minutos**

**4. Agradecimientos/ cierre 10 min**

**Tiempo Total 2 Horas 00 minutos**

**Guía de Tópicos**

| **Tema** | **Estímulos** | **Duración** |
| --- | --- | --- |
| **Sección I. Introducción**   - Introducción del Moderador - Agradecimiento a los participantes por su tiempo y contribución - Explicar en qué consiste el estudio “En esta parte se hablará sobre la compra y elección de alimentos pre empacados y bebidas envasada, particularmente para poder obtener las ideas o pensamientos sobre los criterios que se consideran al momento de elegir productos” - Explicar que se grabarán las opiniones de los participantes, resaltando que la información y opiniones que proveen serán para uso interno y con fines de elaborar un reporte global de opiniones sin identificar a los respondientes - Explicar la importancia de dar opiniones honestas, además que toda información que se aborde durante la sesión será tratada de manera confidencial - Obtención del consentimiento grupal informado | Ninguno | 5 min |
| **Sección II. Percepciones en relación al etiquetado frontal y la selección de alimentos**   - Comportamientos: Productos pre empacados y bebidas envasadas que más adquieren en tiendas de conveniencia o abarrotes   - Marcas / productos más mencionados   - Qué los hace elegirlos   - Qué toman en cuenta para preferir una marca sobre otra - Actitudes: Qué tan relevante resulta la información del producto al momento de comprar   - Qué información sí es relevante   - Qué información no lo es o pasa desapercibida - Conocimientos: Conocimiento general del etiquetado frontal de un producto en relación a información nutrimental   - Se sabe de su ubicación   - Se llega a leer dicha información   - Es clara y útil la información - Productos más/menos asociados con el etiquetado frontal y percepciones del etiquetado frontal sobre estos productos   - Salud   *Nota 1:* *Mostrar dos o tres empaques de productos reales disponibles en el mercado elegidos al azar y pedir que ayuden a señalizar y ejemplificar los argumentos que se han venido exponiendo al respecto*   - Reacciones generales: afectivas y racionales - Qué comunican o transmiten estas etiquetas existentes hoy día en el mercado - Claridad, comprensión, credibilidad, resultantes para el organismo - Qué SÍ y qué NO resulta relevante y útil en materia de etiquetado frontal a partir de lo observado en los empaques muestra que hemos elegido al azar? | Dos o tres empaques de productos reales, al azar, para evaluar sus etiquetados frontales | 15 mins |
| **SECCIÓN III. Evaluación aceptabilidad y comprensión de Etiquetados**  *Nota 2:* *En forma monádica se irán mostrando una a una las diversas alternativas de etiquetado frontal ampliadas. El orden de exhibición será rotado en cada sesión*  ***III.I Alternativas Ampliadas***  *Nota 3:* *En este momento, el moderador selecciona la primera de las alternativas ampliadas del etiquetado frontal a explorar.*   - Reacciones generales - Aceptabilidad   - Gusto     - Elementos de gusto de la etiqueta   - Atractivo     - Confianza percibida en la etiqueta   - Carga cognitiva percibida     - Percepción de defectos de formato que dificultan la comprensión - Comprensión objetiva   - Habilidad para comprender los elementos de diseño   - Habilidad para comprender el mensaje central - Utilidad   - Utilidad práctica   - Relevancia motivacional   - Mensaje para la acción: ¿Qué tipo de acción me invita a tomar a partir de la información que recibo?     - Espontáneo     - Ayudado: controlar mi ingesta, preferir un producto sobre otro, inhibir el consumo, etc. - Elementos gráficos y de lenguaje que facilitan la comprensión del mensaje orientativo hacia la selección de alimentos saludables   - Tamaño de los textos   - Información   - Tipografía   - Colores   - Iconografía   ***III.II Tamaño real***  *Nota 4:* *Se muestra ahora el mismo etiquetado, pero ahora de tamaño real en los productos físicos, con los tres productos con un gradiente de calidad nutrimental (desde el más saludable hasta el menos saludable)*   - Reacciones generales - Aceptabilidad   - Gusto     - Gusto por la etiqueta en la cara frontal del empaque   - Atractivo     - Confianza percibida en la etiqueta     - Facilidad de identificación de la etiqueta en el empaque   - Carga cognitiva percibida     - Percepción de defectos de formato que dificultan la comprensión - Comprensión objetiva   - Habilidad para comprender los elementos de diseño   - Habilidad para comprender el mensaje central. Ejemplo: ¿Este etiquetado les transmite que este (según corresponda) producto es el más saludable y este otro (según corresponda) es el menos saludable de los tres?   ***III.III Entorno Competitivo***  *Nota 5:* *Se descubre ahora el poster del anaquel con una variedad de productos alimenticios con el etiquetado frontal*   - Reacciones generales - Aceptabilidad   - Gusto por tener la etiqueta en la cara frontal del empaque   - Atractivo     - Confianza percibida en la etiqueta     - Facilidad de identificación de la etiqueta en el empaque en comparación con otras marcas competidoras   - Carga cognitiva percibida     - Percepción de defectos de formato que dificultan la comprensión - Comprensión objetiva   - Habilidad para comprender los elementos de diseño   - Habilidad para comprender el mensaje central. | Alternativas ampliadas del etiquetado frontal  Productos físicos con etiquetado en tamaño real  Poster de productos con etiquetado frontal ubicados en anaqueles  Mapping de posicionamiento del diseño | 60 min  (12 min por alternativa de etiquetado frontal) |
| **Sección IV. Agradecimientos**  *Nota 9. Se agradece a los entrevistados y hace el cierre correspondiente entregando el incentivo.* | Incentivos |  |

**EVALUATION OF COMPRENSION AND ACE P TABILITY OF FRONT LABELING SYSTEMS**

**Topic guide for the moderator**

**Central Purpose**

***Remember that the main purpose of this study is to help consumers, through frontal labeling on pre-packaged food products, to make healthier food choices***

**Topics**

The following guide of topics provides an overview of the areas that will be covered through the exploration and testing of the various alternatives of frontal labeling . This guide is not intended to be a list of questions. The session can flow freely, the moderator will seek to capture the reactions, thoughts and spontaneous speech as it arises , ensuring that all the key parts were covered

An average of 3 to 5 alternatives frontal labeling examine to n during focus groups , they will be shown monadically, so to leatoria and seeking an order different in each group to give option that most of them have the opportunity to appear in first position .

The flow of the groups can be summarized as follows:

**1. Introduction 5 min**

**2. Choice of pre-packaged food 15 min**

**30 minutes**

**3. Acceptability assessment and understanding of Labeling 90 min**

**12 minutos**

**4. Acknowledgments / closing 10 min**

**Total Time 2 Hour s 0 0 minute s**

**Topics Guide**

| **Topic** | **Stimuli** | **Duration** |
| --- | --- | --- |
| **Section I. Introduction**   - Moderator Introduction - Thanks to the participants for their time and contribution - Explain what the study consists of "This part will talk about the purchase and choice of pre-packaged foods and packaged beverages, particularly in order to get ideas or thoughts about the criteria considered when choosing products" - Explain that the opinions of the participants will be recorded, highlighting that the information and opinions they provide will be for internal use and for the purpose of preparing a global opinion report without identifying the respondents - Explain the importance of giving honest opinions, and that all information addressed during the session will be treated confidentially. - Obtaining informed group consent | None | 5 min |
| **Section II Perceptions regarding frontal labeling and food selection**     - Behaviors: Pre-packaged products and packaged beverages that are most purchased at convenience stores or grocery stores   - Most mentioned brands / products   - What makes them choose them   - What do they take into account to prefer one brand over another - Attitudes: How relevant is the product information at the time of purchase   - What information is relevant   - What information is not or goes unnoticed - Knowledge: General knowledge of the frontal labeling of a product in relation to nutritional information   - It is known of its location   - You get to read this information   - The information is clear and useful. - Products more / less associated with frontal labeling and perceptions of frontal labeling on these products   - Health     *Note 1: Show two or three packages of real products available in the market chosen at random and ask them to help signal and exemplify the arguments that have been presented in this regard*           - General reactions: affective and rational - What do these existing labels communicate or transmit on the market today? - Clarity, understanding, credibility, resulting for the organism - What YES and what is NOT relevant and useful in terms of frontal labeling from what is observed in the packaging shows that we have chosen at random? | Two or three packages of real products , at random, to evaluate their front labels | 15 mins |
| **SECTION III Acceptability assessment and understanding of Labeling**    *Note 2: In a monadic way, the various extended frontal labeling alternatives will be shown one by one . The display order will be rotated in each session*    ***III.I Extended Alternatives***  *Note 3: At this point, the moderator selects the first of the alternative s extended s front labeling exploring.*   - General reactions - Acceptability   - Taste     - Taste elements of the label   - Attractive     - Perceived confidence in the label   - Perceived cognitive load     - Perception of format defects that make understanding difficult - Objective understanding   - Ability to understand design elements   - Ability to understand the central message - Utility   - Practical utility   - Motivational relevance   - Message for action: What kind of action invites me to take from the information I receive?     - Spontaneous     - Helped: control my intake, prefer one product over another, inhibit consumption, etc. - Graphic and language elements that facilitate the understanding of the orientation message towards the selection of healthy foods   - Text size   - information   - Typography   - Colors   - Iconography     ***III.II Actual size***  *Note 4: The same labeling is now shown, but now of real size in physical products, with the three products with a gradient of nutritional quality (from the healthiest to the least healthy)*   - General reactions - Acceptability   - Taste     - Taste for the label on the front side of the packaging   - Attractive     - Perceived confidence in the label     - Ease of identification of the label on the packaging   - Perceived cognitive load     - Perception of format defects that make understanding difficult - Objective understanding   - Ability to understand design elements   - Ability to understand the central message. Example: Does this labeling convey that this (as appropriate) product is the healthiest and this other (as appropriate) is the least healthy of the three?     ***III.III Competitive Environment***  *Note 5: The shelf poster with a variety of food products with front labeling is now discovered*   - General reactions - Acceptability   - Nice to have the label on the front side of the packaging   - Attractive     - Perceived confidence in the label     - Ease of identification of the label on the packaging compared to other competing brands   - Perceived cognitive load     - Perception of format defects that make understanding difficult - Objective understanding   - Ability to understand design elements   - Ability to understand the central message. | Expanded Front Labeling Alternatives  Physical products with real size labeling  Poster of products with frontal labeling located on shelves  Design positioning mapping | 60 min  (12 min per front labeling alternative) |
| **Section I V . Thanks**  *Note 9 . The interviewees are thanked and make the corresponding closing giving the incentive.* | Incentives |  |
