# Supplementary material for: Front-of-pack nutritional labels: Understanding by low- and middle-income Mexican consumers
Source: PLoS One. 2019 Nov 18;14(11):e0225268. doi: 10.1371/journal.pone.0225268 (PMC6860442; doi:10.1371/journal.pone.0225268)
Supplement: S3 File — (DOCX) [file pone.0225268.s006.docx]

**Cuestionario de aceptabilidad y comprensión de los**

**etiquetados nutrimentales frontales existentes**

**FOLIO: _________________________**

**Instrucciones:** Lee el puntos o preguntas a contestar y tacha o subraya la respuesta según corresponda:

**1.- INFORMACIÓN DEMOGRÁFICA**

| - 1. **Sexo:**   0= Femenino 1= Masculino | **1.2.- Edad: (Años cumplidos)** | |
| --- | --- | --- |
| **1.3 Estado Civil:**  0= Soltero  1= casado  2= Divorciado/ separado  3= Unión libre  4= Viudo | **1.4.- Ocupación:**  0= Sin empleo  1= Estudiante  2= Hogar/ casa  3= Doméstico  4= Empleado  5= Comerciante  6= Otro: _____________ | |
| **1.5 Código Postal:** | **1.6.- Algún médico, enfermera, nutriólogo le ha dicho usted que padece o ha padecido de: (Marca con una “x”)**  a) Diabetes  b) Hipertensión/ presión alta  c) Sobrepeso  d) Obesidad  e) Colesterol alto  f) Triglicéridos | |
| **1.7.- Ultimo nivel de educación (último grado de estudios que terminaste):**  0= Ninguna  1= Primaria  2= Secundaria  3= Superior  4= Licenciatura  5= Posgrado | **1.8.- ¿De qué material es la mayor parte del techo de su vivienda?**  0= Material de deshecho  1= Lámina de cartón  2= Lámina metálica  3= Lámina de asbesto  4= Palma o paja  5= Madera o tejamanil  6= Terrado con viguería  7= Teja  8= Losa de concreto o viguetas con bovedilla | |
| **1.9.- ¿De qué material es la mayor parte de las paredes o muros su vivienda?**  0= Material de deshecho  1=Lámina de cartón  2= Carrizo, bambú o palma  3= Embarro, bajareque o paja  4= Madera  5= Adobe  6= Tabique  7= Otro: ___________________________ | **1.10 ¿Cómo obtienes el agua?**  0= Agua entubada dentro de la cocina o baño  1= Agua entubada fuera de la vivienda, pero dentro del terreno  2= Agua entubada de llave o pública  3= Agua de pozo o noria  4= Agua de manantial, río o lago  5= Agua de pipa  6= Otro: ___________________ | |
| **1.11.-**  ***Marca con una “X”* los objetos con los que cuenta en su casa (que funcionen).**  **Aquellos con los que no cuentas, deja el espacio en blanco** | a) Teléfono Fijo | |
|  | b) Automóvil |  |
|  | c) Motocicleta/ motoneta |  |
|  | d) Computadora/ laptop/ Tablet |  |
|  | e) Televisión |  |
|  | f) Servicio de TV |  |
|  | g) Radio |  |
|  | h) Modulares, consola, plancha |  |
|  | i) Plancha |  |
|  | j) Aspiradora |  |
|  | k) Horno de microondas |  |
|  | l) Teléfono celular |  |
|  | m) Acceso de internet |  |
|  | n) Licuadora |  |
|  | o) Refrigerador |  |
|  | p) Estufa de gas |  |
|  | q) Lavadora o secadora |  |
|  | r) Calentador de agua |  |

**2.- PERCEPCIÓN DE ETIQUETADO**

**Instrucciones:** Lee la pregunta y tacha la respuesta que creas correcta (selecciona solo una opción de respuesta):

| **PREGUNTA** |
| --- |
| **2.1.- ¿Cuántas calorías considera usted que una persona adulta debe consumir en promedio al día? Por favor, para responder a esta pregunta, imagine una persona sana de su misma edad y género.**  0) Menos de 500 calorías 1) De 500 a 1,000 calorías 2) De 1,001 a 1,500 calorías  3) De 1,501 a 2,000 calorías 4) De 2,001 a 3,000 calorías 5) De 3,001 a 4,000 calorías  6) Más de 4,000 calorías 7) No sé |
| **2.2.- ¿Usted sabe si los alimentos empacados y las bebidas embotelladas tienen una etiqueta nutrimental frente al empaque?**  0) No--------------**PASE A LA PREGUNTA 2.7**  1) Si---------------**CONTINUE**  2) No sé ---------**PASE A LA PREGUNTA 2.7** |
| **2.3.- ¿Usted lee la etiqueta nutrimental de los alimentos empacados y las bebidas embotelladas que compra?**  0) No 1) Si 2) A veces 3) No sé |
| **2.4.- ¿Cuánto tiempo en promedio le dedicas a leer la etiqueta frente al empaque?**  0) No los veo 1) Menos de 30 segundos 2) De 30 a 59 segundos 3) Entre 1 y 4 minutos  4) Más de 4 minutos 5) No tomo el tiempo 6) No sé |
| **2.5.- ¿Qué tan comprensible es la información nutricional en la etiqueta que se encuentra al frente de los empaques?**  0) Nada comprensible 1) Poco comprensible 2) Algo comprensible  3) Muy comprensible 4) No sé |
| **2.6.- Cuando compra alimentos empacados y/ o bebidas embotelladas, ¿Con que frecuencia elige un producto por la información de los empaques?**  0) Nunca 1) Casi nunca 2) A veces 3) Casi siempre 4) Siempre 5) No sé |
| **2.7.- ¿Por qué razón nunca o casi nunca utiliza las etiquetas nutrimentales frente al empaque de los alimentos empacados y bebidas embotelladas?**  0) No tiene tiempo 1) No le interesa 2) No le es visible 3) No confía en ellas  4) No le entiende 5) No las considera de utilidad 6) Otro (Escribe aquí): _________ |
| **2.8.- ¿Por qué razón a veces o casi siempre utilizarías las etiquetas nutrimentales de los alimentos empacados y bebidas embotelladas?**  0) Por salud/ enfermedad/ nutrición 1) Porque llevo una dieta o régimen de alimentación  2) Porque le interesa conocer 3) Porque están dirigidos a bebés, niños, embarazadas o  adultos mayores  4) Para revisar la calidad del producto 5) Por elegir un producto específico (vegano, sin gluten,  orgánico, etc.)  6) Otra (Escribe aquí): _______________ |

**Acceptability questionnaire and understanding of existing frontal nutrient labeling**

**ID: _________________________**

**Instructions:**Read the point s or questions to answer and cross out or underline the answer as appropriate:

**1.- DEMOGRAPHIC INFORMATION**

| **1.1- Sex:**    0 = Female 1 = Male | **1.2.- Age: (Years completed)** | |
| --- | --- | --- |
| **1.3 Marital Status :**    0 = Single  1 = married  2 = Divorced / separated  3 = Free union  4 = Widower | **1.4.- Occupation:**  0 = No employment  1 = Student  2 = Home / house  3 = Domestic  4 = Employee  5 = Merchant  6 = Other: _____________ | |
| **1.5 Postal Code :** | **1.6.- Some doctor, nurse, nutritionist has told you that you have or have suffered from: (Mark with an “x”)**  a) Diabetes  b) Hypertension / high pressure  c) Overweight  d) Obesity  e) High cholesterol  f) Triglycerides | |
| **1.7.- Last level of education (last degree of studies you finished):**    0 = None  1 = Primary  2 = Secondary  3 = Superior  4 = Bachelor  5 = Postgraduate | **1.8.- What material is most of the roof of your home?**  0 = Waste material  1 = Cardboard sheet  2 = Metallic foil  3 = Asbestos Sheet  4 = Palm or straw  5 = Wood or tejamanil  6 = Roof with viguería  7 = Tile  8 = Concrete slab or joists with vault | |
| **1.9.- What material is most of the walls or walls your home?**    0 = Waste material  1 = Cardboard sheet  2 = Carrizo, bamboo or palm  3 = Embarro, bajareque or straw  4 = Wood  5 = Adobe  6 = Septum  7 = Other: ___________________________ | **1.10 How do you get water?**    0 = Water piped inside the kitchen or bathroom  1 = Piped water outside the house, but inside the land  2 = Piped or public tapped water  3 = Well water or ferris wheel  4 = Spring, river or lake water  5 = Pipe water  6 = Other: ___________________ | |
| **1.1 1 .- *M ark with an "X"*objects with which account at home (that work).**      **Those you don't have, leave blank** | a) Landline | |
|  | b) Automobile |  |
|  | c) Motorcycle / scooter |  |
|  | d) Computer / laptop / Tablet |  |
|  | e) Television |  |
|  | f) Inspection io TV |  |
|  | g) Radio |  |
|  | h) Modular, console, iron |  |
|  | i) Iron |  |
|  | j) Vacuum cleaner |  |
|  | k) Microwave oven |  |
|  | l) Cell phone |  |
|  | m) Internet access |  |
|  | n) Blender |  |
|  | o) Refrigerator |  |
|  | p) Gas stove |  |
|  | q) Washer or dryer |  |
|  | r) Water heater |  |

**2.- PERCEPTION OF LABELING**

**Instructions:**Read the question and cross out the answer you think is correct (select only one answer option) :

| **QUESTION** |
| --- |
| **2.1.- How many calories do you think an adult should consume on average per day? Please, to answer this question, imagine a healthy person of the same age and gender.**    0) Less than 500 calories 1) 500 to 1,000 calories 2) 1,001 to 1,500 calories    3) From 1,501 to 2,000 calories 4) From 2,001 to 3,000 calories 5) From 3,001 to 4,000 calories                                          6) More than 4,000 calories 7) I don't know |
| **2.2.- Do you know if packaged foods and bottled drinks have a nutritional label against the packaging?**  0) No ------------- - **SKIP TO QUESTION 2.7**  1) Yes ------------- - **CONTINUE**  2) I don't know --------- **SKIP TO QUESTION 2.7** |
| **2.3.- Do you read the nutritional label of packaged foods and bottled beverages you buy?**    0) No 1) Yes 2) Sometimes 3) I don't know |
| **2.4.- How much time on average do you spend reading the label in front of the packaging?**    0) I don't see them 1) Less than 30 seconds 2) 30 to 59 seconds 3) Between 1 and 4 minutes                    4) More than 4 minutes 5) I don't take the time 6) I don't know |
| **2.5.- How understandable is the nutritional information on the label on the front of the packaging?**        0) Nothing understandable 1) Little understandable 2) Something understandable                                             3) Very understandable 4) I don't know |
| **2.6.- When you buy packaged food and / or bottled beverages, how often do you choose a product for the information on the packaging?**    0) Never 1) Almost never 2) Sometimes 3) Almost always 4) Always 5) I don't know |
| **2.7.- For what reason do you never or almost never use nutritional labels against the packaging of packaged foods and bottled beverages?**    0) He has no time 1) He is not interested 2) He is not visible 3) He does not trust them    4) Do not understand 5) Do not consider them useful 6) Other (Write here): _________ |
| **2.8.- Why would you sometimes or almost always use the nutritional labels of packaged foods and bottled beverages?**    0) For health / illness / nutrition 1) Because I have a diet or diet    2) Because you are interested in knowing 3) Because they are aimed at babies, children, pregnant or                                                                                                             older adults    4) To check product quality 5) By choosing a specific product (vegan, gluten free,                                                                                                                 organic, etc.) |
