# Supplementary material for: Front-of-pack nutritional labels: Understanding by low- and middle-income Mexican consumers
Source: PLoS One. 2019 Nov 18;14(11):e0225268. doi: 10.1371/journal.pone.0225268 (PMC6860442; doi:10.1371/journal.pone.0225268)
Supplement: S4 File — (DOCX) [file pone.0225268.s007.docx]

Coding guide focus groups

Project: **Front nutrition labeling of industrialized foods for Mexican consumers**

| **Category :**Perceptions of sctual labeling and food choices . |
| --- |
| **Description :**General reactions to current frontal labeling in different age groups and socioeconomic level citing different support materials . |

| **Properties** | **Description** | **Dimensions** | **Description** | **Specificities** | **Description** | **Specificities** |
| --- | --- | --- | --- | --- | --- | --- |
| Behaviors | Packaged products and beverages that you buy most at convenience stores or grocery stores | Brands / Products | Mention of trade names or products that perform to encirclement towards the products and thus deduce the selection | Age differences | Qualities, characteristics and / or reasons for choosing a product . | - Teens from 13 to 15 years old  - Young adults from 21 to 23 years old  - Family parents with children between 3 and 12 years old  - Moms with children between 3 and 12 years old  - Adults over 55 to 70 years |
|  |  | Choice | Reasons why they consider selecting between different products . | Socioeconomic level | Individual labor and economic preparation that allows a selection of a product. | - Low  - Medium |
|  |  | Preference | Reasons, reasons or circumstances why it considers selecting a product or brand compared with OTR to . | Support materials | Understanding the current nutritional label with the use of the different materials used | - Board extended  - Physical products  - Anaquel |
| Attitudes | Relevance and / or consideration of information for decision making in the purchase of a product. | Importance of the information | Relevance of the information considered when making purchasing decisions for an industrialized product |  |  |  |
|  |  | Irrelevant information | Information unnoticed by the consumer for the purchase of an industrialized product |  |  |  |
|  |  |  |  |  |  |  |
| Knowledge | Knowledge s General is the front labeling current in relation to the nutrition information . | Label location | Knowledge of the current label positioning |  |  |  |
|  |  | Label reading | Reasons and / or reasons why people read the current nutritional label. |  |  |  |
|  |  | Clarity and usefulness of the label | Understanding and benefit of the current nutritional label |  |  |  |

| **Category.**Acceptability |
| --- |
| **Description. F**avorable characteristics that meet the nutritional front labeling that is determined by several factors including the liking for the label, how attractive it is and the perceived cognitive load of the label . |

| **Properties** | **Description** | **Dimensions** | **Description** | **Specificities** | **Description** | **Specificities** |
| --- | --- | --- | --- | --- | --- | --- |
| Liking | Elements characterizing the label and that are of gust or l consumer | Age differences | Qualities, characteristics and / or reasons for choosing a product . | - Teens from 13 to 15 years old  - Young adults from 21 to 23 years old  - Family parents with children between 3 and 12 years old  - Moms with children between 3 and 12 years old  - Adults over 55 to 70 years | - |  |
| Attractive | Perceived confidence or ease of identification regarding nutritional front labels | Socioeconomic level | Individual labor and economic preparation that allows a selection of a product. | - Low  - Medium | - |  |
| Perceived cognitive load | Default perceptions of each nutrient front label format that make understanding difficult | Support materials | Understanding the current nutritional label with the use of the different materials used | - Board extended  - Physical products  - Anaquel | - |  |
|  |  | Front Nutrition Labels | General reactions of 6 existing nutritional labels | - Healthy Star Index (CO)  - Healthy Option (NA)  - 5-Nutritional Color Labeling (RO)  - Black Warning (BN)  -Red Warning (BN-R)  - Multiple Traffic Light (VE) | - |  |

| **Category.**Objective Understanding |
| --- |
| **Description.**Information understood by consumers consistent with information provided by the fron of packe labelling as more accurate measurement, not susceptible . |

| **Properties** | **Description** | **Dimensions** | **Description** | **Specificities** | **Description** | **Specificities** |
| --- | --- | --- | --- | --- | --- | --- |
| Design | Consumer skills to understand graphic and language elements of different nutrient label formats | Graphic and language elements | Ability of the consumer to understand elements and characteristics of designs or formats of different nutritional front labels | Size | - | Age difference |
|  |  |  |  | information | - | Socioeconomic level |
|  |  |  |  | Typography | - | Support materials |
|  |  |  |  | Colors | - | Front Nutrition Labels |
|  |  |  |  | Iconography | - | - |
|  |  |  |  | Front nutrition labeling | - | - |
| Central message | Facility consumer to understand the message focuses l a label that lead to the selection of another product. | Utility | Consumer ability to understand message focuses l different nutritional front labels | Practicality | - | - |
|  |  |  |  | Motivational Relevance | - | - |
|  |  |  |  | Message for action | - | - |
