# Supplementary material for: Front-of-pack nutritional labels: Understanding by low- and middle-income Mexican consumers
Source: PLoS One. 2019 Nov 18;14(11):e0225268. doi: 10.1371/journal.pone.0225268 (PMC6860442; doi:10.1371/journal.pone.0225268)
Supplement: S5 File — (DOCX) [file pone.0225268.s008.docx]

***GDA perception-food choices***

| **CODES** | | **Adolescents between 13 and 14 years old** | **Young adults between 21 and 23 years old** | **Mothers with children between 3 and 12 years old** | **Parents with children between 3 and 12 years old** | **Seniors between 55 and 70 years old** |
| --- | --- | --- | --- | --- | --- | --- |
| **Behaviors** | **Brands / Products** | 2: In the presentation... M 2: Yes the mark…M 1: What do you look, at the brand in which it is known?  2: No, the unknown too ... B | 2: For its presentation…M 2: You are interested in presentation…M  2: Craving the product ... B 1: Craving and experience…M 2: The taste goes for a specific one and if has a fruit that catches my attention I buy it …B 1: Open, try new products... B 2: I'm hungry and I buy donuts, there are those of Bimbo, a brand that i don't know and those of the store, for the brand you think is better quality, but maybe the store is better ...B | 2: Brand ... M 2: I buy some beans if I liked them and if I don't, try others, and when I know which one I liked, I go back to buy it…M 2: I used to buy from “La Costeña” like this in a can, and now that I tried Isadora, how those beans fascinate me!… B | 2: The Brand… M 1: The visual features, the beautiful aesthetic, the striking thing about the packaging… B | 2: I think that comparison between brands ... M 2: Yes, the colors, their logos ... B 2: Over time you end up with certain brands…B 2: The brand... B 2: The presentation ... M |
|  | **Choice** | 2: Taste…B 2: Price… M 2: The price… B 2: The one I crave the most…B  2: The one that looks tastier…M 2: Be healthy… M | 2: Previous experience…M  2: The taste, the price…M 2: The craving of the moment…M 2: Packaging design ... M 2: And depending on the craving you have, soda or water-…M 1: Quantities  2: If it is bigger ... B | 2: Size…B 2: If can be easily closed or you can take them …B 1: If is easily transportable  2: If it is liquid, for example…B 1: Screw cap.  2: Easy to make in microwave…M 1: Price  2: Yes, price 1: What else?  2: Its packaging…M 2: Be practical, be beautiful…Ml 2: Nutritional information…,M 2: Product image ... M | 2: Taste 2: Price… M 2: Depending on what I like. If you´re craving of some peanut snacks, Sabritas..B | 2: The labels, for example if there´s an image, what does that image reflect to me…M 1: What else do I look at when I´m choosing it?  2: The price ... B 2: The price and the utility it has for us ... B |
|  | **Preference** | 2: Taste  2: Price ... M 2: In the flavor  2: Not because if you are going to buy it how will you know what it tastes like  2: No, but the taste that the packaging shows, if it is orange ... B |  |  | 2: Time, in the morning it can be a coffee. And in the afternoon for the rush of the work, you buy a “Lunchibon” ... B | 2: Like whole wheat bread I prefer white, white bread…M  2: Quality and price more than anything… B |
| **Attitudes** | **Importance of the information** | 1: When you want something healthy, where do you find it?  2: In the market…M  2: In the supermarket…B  2: In a specific store… B | 2: When you're interested, you're looking for it and when you're not interested… B | 2: I noticed that my husband has to go to a nutritionist, I began to look at the products because he needed a certain type of calories ... B 2: Honestly I look at the food when I I'm going to the supermarket. I have a diabetic son and since he has diabetes... B | 2: The nutritional table is for the portion, if you buy a box it comes by portion of what you are going to eat, not by piece, if you take a coca it´s not by the liter, is by milliliters that you are going to take by glass…B  2 : Also the information a while ago asked how many calories you should take on average, I have no idea and even if I say it has a hundred or eight calories if I don't know how many I should eat, it has no point ... M | 2: Especially in quality ... M |
|  | **Relevant information** | 2: Sometimes I try to read them because sometimes I like it and because sometimes I take care of myself, that's why sometimes I read it and buy, for example, all products with calories and fat, I choose them, but there are times that I don´t…M 2: It's how I feel, sometimes I feel like she says, I have to take care of myself, or if you feel bad, I don't know… B |  | 2: No, […] if you drink a glass like this, it has 5% sugar, but in the back it says you have sugar, tracing, sodium ... B 2:Expiry…B 2: Depends on who the food is for, if it's for me I see what I´m consuming, for the child, that they eat well at home, I let them eat junk food, it is the idea I do not buy healthy food in Oxxo ... M | 2: In general, for example “Splenda” is a sugar substitute sachet, I don't like it, I don't like it, when it is an added product I don't buy it…M 2: For packaging, the quantities are all that the packaging brings, is what you will eat, if you buy some chips of this size the label is indicating the portion of the package.… B | 2: What interests me about this table is the sodium content, it´s what causes fluid retention, there are people who are allergic and because they do not read, they can eat something ... M 2: Its expiration date... B |
| **Knowledge** | **Label location** |  |  | 2: There are some ingredients that we don't know what it is, they have their name, I don't know 2: It comes behind the introduction ... B  2: You don't see anything, you don’t understand…B 2: You don't see…B  1: It makes reading difficult what other things we see?  2: I create the color…M  1: Does the color doesn´t help much?  2: No. …M 2: Size…M 2: It says other fats, saturated fats and other fats and here it repeats and says energy 182 kilocalories, now energy per container…M 2: The main problem is very small ... M |  | 2: You need to look for it…M   2: It doesn't looks good …M 2: You can't see it: You forget your glasses and I can't see …B 2: This one is not visible 2: It's clear, it looks like the big one is clear and it looks better, calories…B. 2: I can't see them…B 2: I need a magnifying glass ... B |
|  | **Label reading** | 1: Ok. I have here some products, some are empty or not, see and analyze examples. Already located? ... perfect. Ok let's concentrate on this tablet, perfect, tell me, what do you see there? How do you interpret it? How do you interpret it? Not exactly every data, but what kind of information is giving to me ...  2: Percentage of calories…B  2: Fats …M 2: Cholesterol…M  2: Sodium…M 2: Calories. …M 2: Proteins ... M 1: How much do you see this labeling of nutritional indications ?, Do you do? Do you notice [the labeling]?  2: Sometimes **B**  2: Rather, I believe, for example, when I go to Oxxo to buy, I go where they are, I open it and it would be, it is how you see it and how you already buy it and now ...B | 1: You didn´t tell me if you consider the nutritional table, do you look at it?  2: No…B  1: Why not?  2: Only those who take care of themselves, but almost nobody ...B 1: My question is when we read them, do we understand them? (FOPL)  2:I don´t understand the percentages, you cannot see that it is 100% for the person, it matters weight and everything, how they get that percentage, for an adult or child ... M | 2: More than anything, they have not taught us, in my case, or I have not paid attention to how to read a nutritional table and I can take and start buying the products, that if I know, I would pay a little attention to everything…M  1 : We don't have the culture  2: My grandmother…, we have to buy her bread, she told us it has to be low in calories, in sugar there if we look at it .. M 1: How much do you look at the labels or nutritional tables when you´re choosing a product  2: Due to lack of time, we no longer stop to see what is in the ingredients, we do not know what it is, they have their name, I do not know ... scientist, yes scientist, you are already used to buy, and you don´t notice ... B | 1: Ingredients 6 and 7, with this I already have how are you making your decision trees, how much do we bald and look at the nutritional tables, the others?  2: Very little…B 2: Little…B 2: Nothing ... M | 2: I mean the squares that say: kilocalories, sodium, it is information that I do not know if you have to be a scientist to understand, because if it tells me X percentage in two liters contains 8 portions, 130% but based on what? That is why I cannot make comparisons, if they tell me I can consume as much sugar and as much sodium I already know and compared to this, with what I compare if I have no idea ... M 1: When I look at these things, nutritional tables, terminology I understand or is difficult to understand  2: I do not even notice…B 2: It is difficult to understand ... B |
|  | **Clarity and usefulness of the label** | 1: And how much it would be?  2: like a glass, a quarter…B  1: How much is the portion here?  2: 13.5 grams of a hamburger …B 1: 13.5 of a hamburger is what you have, how much would a hamburger weigh?  2: like a glass…B  1: But how much does it weigh, and it explains in grams, both 13.5 is the serving and is equivalent to that, how much is a serving of a hamburger? For example, that, how much is the portion?  2: 200 GR. …M 2: Well, more or less a meat…M  1: If it will weigh 13 grams.  2: Or maybe 2 ...M  1: Maybe 2 hamburgers weigh 13 grams, that ... And of this how much is the portion, how much it said?  2: Size proportion a piece 11 grams…B  1: A piece would understand that it weighs 11 grams, if this weighs 11 grams a hamburger...  2: Well, it is divided, right?, It is divided by the amount of hamburgers and that would weigh each one ... M | 2: Here it says “a portion of 240 milliliters provides”, 240 milliliters is what contributes, not everything, [it is] like a glass…M 2: This one says of nutrients, how much% it has of nutrients…M 1: Let's see if seeing it bigger it is clear to us:  2: I do not understand the percentages…M  2: You cannot see that it is 100% by the person, it matters weight and all…M  2: And how they get that percentage…M  2: For an adult or child ... M 2: Are very small…M  2: Yes, it comes too small…B  2: Hard to understand…B  2: Not visible…B  2:You are not interested…B 1:What did I understand, they explained to me that it confused me:  2: Below it says sodium and energy as it gives you energy with everything you have…B  2:A basic question is what is saturated fat? What are the other fats? The other sugars? And how do you have energy?...B | 1: Do we understand? are you clear?  2: Yes, they are very clear  2: Sometimes they are clear. You have to make divisions…B  2: Yes, let's see how many I'm going to eat… and energy…B  2: It says 12 hamburgers and then 13.5 you understand are 12 and 13.5 per container…M  2: We no longer understand…M  2 : Per piece…m  1: And then type of fats, yes, it's complicated  2: And if you don't have enough time to interpret what is the use of grabbing this and the other and you don't know anything ... M | 2: Because I don't understand…M  2: I don't understand the same 2: It's complex ... B | 2: It doesn´t attracted my attention, because the pictures are very small, but it started to get my attention because they put it very flat, because they are very tall, those with large functional pictures are more balconeados then now I read all…M 2: I don´t understand why, in a period of time I forgot about this, I played basketball, always doing clinical studies, as long as I don't have it I don't know what is missing or left over that's very important, I note that yes and no, there are labels that are poorly readable and those [should be] discarded…M  2: The labels don´t have the amount you require, you don´t know what can affect you…M   2: I´m worry when it has Aspartame, I understand that are bad for your health…B  2: Also sugars…B 2: Salt ... B |
| ***1, Moderator; 2, Participant; M, Medium Socioeconomic Level; B, Low Socioeconomic Level*** | | | | | | |

**Guidelines Dairy Allowence (GDA)**

| **CODES** | | **Adolescents between 13 and 14 years old** | **Young adults between 21 and 23 years old** | **Mothers with children between 3 and 12 years old** | **Parents with children between 3 and 12 years old** | **Seniors between 55 and 70 years old** |
| --- | --- | --- | --- | --- | --- | --- |
| **Acceptability** | **Taste** | 2: I like them to tell me the calories, some people see them ... B 2: The truth is that everyone knows what she/he eats it is not necessary to decide because I already eat it ... M | 2: If I like what it says I see what it has, but I don't know how much I have to eat ... M | 2: There are some ingredients we don't know what it is, they have their name, I don't know…B 2: It comes behind the introduction ... B  2: You don't see anything, less you understand…B  2: You don't see ... B  1: It makes us difficult reading, what other things do we see?  2: I create the color…M  1: Doesn´t the color help much?  2: No…M  2: Size…M  2: It says other fats, saturated fats and other fats and here it repeats and says energy 182 kilocalories, now energy per container…M 2: The main problem is very small ... M | 2: I don't like it, I really need a measuring cup for this ... B 2: I have to bring a scale or what ... B | 2: It didn´t attract my attention, because the pictures are very small, but it started to get my attention because they put it very flat, because they are very tall, those with large functional pictures are more visible then now I read all…M 2: I don´t understand why, in a period of time I forgot about this, I played basketball, always doing clinical studies, as long as I don't have it I don't know what is missing or left over that's very important, I note that yes and no, there are labels that are poorly readable and those [should be] discarded…M |
|  | **Attractive** | 2: when I enter in a store, I see it and I already buy it ...M 2:I don't really look at the label ... M 2: Me, when I already buy it and I'm on my way I sometimes read it ... M | 2: I would see everything, except the label, the price or the nutritional table does not indicate anything to me… M 2: I notice it when I have nothing to do and see it, but not [affect] in my purchase decision… .M | 2: Lack of time to read and we can't stop so much… B | 2: the truth is that is very small and very confusing ... B 2: if I was diabetic I would need to see the sugar and die ... B |  |
|  | **Cognitive Charge Perceived** | 1: What does the portion tell me? How much would the portion be? 2: the portion size is one piece of 11 grams. It is divided from the total and we already get how much is worth of calories…M 1: Is it easy to understand? 2: No, it depends on the product, it is easy in milk, in a hamburger it is not so easy ... B 2: I think it is interpreted to see what you are buying what is most says ... M | 2: I don't see it because I don't understand the language they use ... B. 2: Maybe is something cultural because I've never taken care of myself and I'm not in control of what do I eat… B | 2: It is very complicated, what it shows in the sodium and fat package and so ... B | 2: Sometimes I do not understand because we are not a nutritionist ... M 2: you have to do the calculation and the nutritional table comes by the portion, if you buy a box it comes by the portion and not by the complete packaging ... B | 2: On the labels you don't know how much do you need, you don't know what can affect you...B   2: I´m worry when it has Aspartame, I understand that are bad for your health…B 2: Also sugars…B  2: Salt ... B |
| **Objective understanding** | **Design** | 2: They have the calories, sodium, percentages and amounts of grams of each ... B 2: Those with calories, sodium and that .. More or less I understand ... M | 2: I do not notice because it is very small, it is not in sight ... B 2: I think that is the percentage that I have to add, although they are over past are more than 100% added ... B | 2: the truth is that I almost don't understand the percentages and the circles that it has… B | 2: There you see the label in a big way, but in this product that I have it is not even possible to see ... M 2: I like more what it says other things if it is healthy or so, but I would not notice the label ... B | 2: I was not attracted to attention and more because the pictures are very small, but it started to get my attention because they put it is very flat, because they are very tall, those with large functional pictures are more visible then now I read all…B 2: I didn't understand why, in a period of time I forgot about this, I did a lot of basketball, always doing clinical studies, as long as I don't have it I don't know what is missing or left over that's very important, when I'm in That, I note that yes and no, there are labels that are poorly readable and those discarded…M |
|  | **Central Message** | 1: Ok. I have here some products examples, some are empty or not, see and analyze examples. Already located? ... perfect. Ok let's concentrate on this tablet, perfect, tell me, what do you see there? How do they interpret it? How do they interpret it? Not exactly every data, but what kind of information is it giving me ...  2: Percentage of calorie…M  2: Fats…M  2: Cholesterol…M  2: Sodium…M  2: Calories…M  2: Proteins ... M 1: How much do you notice in this labeling of nutritional indications ?, do you notice it? Don´t you look at it?  2: Sometimes…B  2: Rather, I believe, for example, when I go to Oxxo to buy which is where they are I go to where they are, I open it and it would be, it is how you see it and how you already buy it and now ...B  1 : Sideways? What will it depend on when and when not? ... B | 2: Here it says a portion of 240 milliliters provides, 240 milliliters is such a thing more or less ... and it is what contributes not everything like a glass…B 2: This one says of nutrients, how much% it has of nutrients ... B 1 :  Let's see if seeing it bigger is clear to us: 2: I don't understand the percentages ... M  2: You can't see that it is 100% for the person matters weight and all…M  2: And how does they get that percentage ... M  2 : For an adult or child ... M 2: They are very small ... M  2: yes it comes too small ... M  2: Difficult to understand ... B  2: Not very visible ... B  2 : You are not interested ... M 1: What did I understand that they explained to me that it confused me:  2: Below it says sodium and energy as it gives you energy with everything it has ... M  2: A basic question is what is it? saturated fat? What are the other fats? The other sugars? And how do you have energy? ... B | 1: Do you understand? are you clear?  2: Yes, they are very clear…M  2: Sometimes they are clear You have to make divisions…M  2: Yes, let's see how much I'm going to eat, and energy…M  2: It says 12 hamburgers and then 13.5 you understand are 12 and 13.5 per container…M  2: We no longer understand…M  2 : Per …piece  1: And then type of fats, if it's complicated  2: And if you don't have enough time to interpret what is the use of grabbing this and the other and you don't know anything ... M 2: I do understand him but it is because I already went with a nutritionist and he explained to us ... B | 2: I don't know how many calories I can consume, because I have no idea about that ... M. 2: that I add it or show it… B. 2: say calories, is it calories or kilocalories? ... B. 2: this is supposed to contribute to your body and I need the daily percentages ... M |  |
|  | **Graphic and language elements** | 1: How clear is this table? 2: No, it is not so clear, a zero, it is not known how many calories I should eat ... B I would look a little more at the color and presentation already shown on the shelf ... I would not look at the nutritional table ... I am not interested in seeing much nutritional information ... B. 1: With just the table in front could you select between one and the other? 2:No, I don´t ... M | 2: We don't understand the percentage ... M 2: I don't know how much I should eat if that or less ... M 2: I'd look at those percentages because it's high, but I don't know how much ... M. 2: To begin with we would have to define what a calorie is, sodium, saturated fat ... B 2: above all and the problem I have is that I would always have to know where they get that percentage ... B |  | 2: We don't have the references or the parameters, we don't know what it has ... M 2: I really don't understand what it says in one, I don't know what it is about, saturated fat, sodium and sugar, but all the sugar? ... B | 2: I mean the squares that say kilocalories, sodium, it is an information I do not know if you have to be a scientist to understand him, because if he tells me X percentage in two liters contains 8 portions 130% but based on what? That is why I cannot make comparisons, if they told me I can consume as much sugar as much, so much sodium I already know and compared to this, with what I compare if I have no idea ... M 1: How much do I look at these things, nutritional tables, terminology I understand or is difficult to understand  2: I do not even notice ... B  2: It is difficult to understand ... B |
|  | **Utility** | 1: And would it be like how much of that?  2: like a glass, a quarter  1: How much is the portion here?  2: 13.5 grams of a hamburger  1: 13.5 of a hamburger is what you have, how much would a hamburger weigh?  2: like a glass… B  1: But how much does it weigh, and since it explains in grams, both 13.5 is the portion and is equivalent to that, how much is a portion of a hamburger? For example, that, how much is the portion?  2: 200 GR ... B  2: Well, more or less a meat  1: If it will weigh 13 grams.  2: Or maybe 2 ... B  1: Maybe 2 hamburgers weigh 13 grams, that ... And of this how much is the portion how much it said?  2: Size proportion a piece 11 grams...M  1: A piece would understand that it weighs 11 grams, if this little girl weighs 11 grams a hamburger ...  2: Well, it is divided, right?, It is divided by the amount of hamburgers and that would weigh each one ... M | 1: Do you know what it says there? 2: yes, calories, saturated fat, sodium ... M. 1: do you know that it is saturated fat? 2: no ... good is good fat, right? ... M 2: yes, or unhealthy fat, I don't know ... M | 2: More than anything they have not taught us in my case or I have not paid attention to how to read a label and I can grab and start buying the products, that if I taught me I would pay a little attention to each thing…M  1: We do not have the culture  2: My grandmother, we have to buy her bread, she told us it has to be low in calories, in sugar now we look ... M 1: How much do you look at the labels or nutritional tables when choosing a product  2: Due to lack of time, we don't stop to see what's in the ingredients, we don't know what it is, they have their name, I don't know ... scientist, yes scientist, you're used to buying and you don't notice ... B | 2: it is necessary that they say for the envelope it has… M 2: Maybe it is not convenient for you since you see or you will want to buy it… M 2: according to me, I drink sodium and it says 73% I suppose I need 72% of salts that Do you have the product? ... B |  |
| ***1, Moderator; 2, Participant; M, Medium Socioeconomic Level; B, Low Socioeconomic Level*** | | | | | | |

**Multiple Traffic Light *(MTL)***

| **CODES** | | **Adolescents between 13 and 14 years old** | **Young adults between 21 and 23 years old** | **Mothers with children between 3 and 12 years old** | **Parents with children between 3 and 12 years old** | **Seniors between 55 and 70 years old** |
| --- | --- | --- | --- | --- | --- | --- |
| **Acceptability** | **Taste** | 1: How do you see this label? Is it clear, do you understand it, don't you understand it, do you like it?  2: I don't understand it…B  2: The same question is how much is low and how much is high ... M What does it tell you? …B  2: Red is avoid... B |  | 2: Green…B  2. It is healthy…B  2. It does not contain fat…B  2. It is not transparent that does not contain fat, has no logo ... M |  | 2: By the colors we identify more, although we do not reach to read, and we already see what is very high in red, for example, see if I am interested in sugar or sodium…B 1: We will assume that instead of this stamp is this stamp:  2: I keep the one here…M  2: I like this  two more…M 2: I like it… M |
|  | **Attractive** | 1: I want you to tell me, How do they see it? Do you see it?  2: Go on, yes…M  1: Why yes?  2: Because it says, for example, you are going to buy a juice, there it says low sugar and you say I'm going to get fat and you don't buy it anymore. The colors tell me something does not tell me something tell me? Tell me to see? …M  2: The red color speaks to me like ...M  2: As a warning ...M  2: Danger ...  2: It's like a traffic light ...M  1: The green color that tells me?  2: What is good ...M  2: Recommended ... M 1: Or, for example, in the Ades as it would look, am I clear more or less? How does it look there?  2: Nice ...B  2: Easy to understand, I don't need more ...B  2: It's flashy ... B | 1: VE How do you see it?  2: It looks easier to understand…M  1: How are you telling me?  2: High in sugar so I don't buy it…M  1: It tells you that it's high and low  VE (MTL). Let's see?  2: It's understandable…B  2: It´s not too had? 2: It's a traffic light type 2: Being able to make a difference between the best and the worst, the worst has too much sugar and the best is low in fat, and the bottom, not so good , but almost no sugar and the worst has too much sugar ... B | 2. I like it more than the other of the little balls…M  2. It's the same idea as the black one…M  2. I like that one…M  2. It looks colorful here…M  2. clearer ... M 2: When I saw that one I recognized the colors of the traffic light…B  1: Traffic light.  2: For my very specific eye contact, as specifically sugar…B |  | 2: It looks easier to understand…B   1: What is it communicating to me?  2: What is high in sugar…B  2: Low in sodium ... B 1: What are they indicating?  2: Even if it is an alert…M  2: Like a traffic light…M  2: That you can go, like the traffic light, yellow is the preventive one and red the care… M |
|  | **Cognitive Charge Perceived** |  | 2: But it doesn't detail figures…M  2: What do you call high in sugar? Maybe I 30% and you 50 ... M |  |  |  |
| **Objective understanding** | **Design** | 1: What is the intention or what does this label want to communicate about the product? m 2: What is the healthy level…M  2: What´s the contain, food…M  2: And how much…B  1: How is the amount expressed?  2: High, medium and low…B  2: One has less and the other more ... B | 2: The traffic light is common, we always see it, the same cell phone ... M | 1: If my bars are green, what are you telling me?  2: Yes…B   2: Low sodium…B  1: Do you mean?  2: It's healthy…B  1: It's healthy. And if yellow colors predominate, what are you telling me?  2: It's more or less…M  1: If the red ones predominate  2: It's not right… M |  |  |
|  | **Central Message** | 1: Here you understand, it's easy to understand. What else is this telling you? If you see it on the label?  2: Sugar is high, sodium low and…B already ...  1: Is it clear?  2: Yes ... B | 1: What does it tell me?  2: As alerts…M  1: Alert What color?  2: The red…M  1: The green that tells me?  2: Okay…M  2: Exact…M  1: And the orange one this:  2: Midpoint…M  2: Be careful… M | 1: Why is it going well?  2: It has 2 greens…M  1: Two greens and there are no reds 2: And size  1: How do they classify it so fast in health, where is it?  2: In between…B  2: Yes in between me too…B  2: In the middle because it has red and green…B  2: It has a lot of sugar…B  2; In the middle 1: How are you? …M  2: It's healthier…M  2: Higher…M  2: Two in favor…M  2: Two green and two yellow…M  1: And not red…M  2: Exactly…M  2: But the ideal thing would be for him to say he has no sodium ... M | 1: What do you say it says?  2: It has everything that I lack for those, it has a traffic light…B  2: The high low medium…B 1: Draws Do you mean that there are drinks that have more red?  2: Yes…B  2: Yes…B  2: More redder greens…B  2: Drinks that are not so healthy ...B 2: In the food chain they also handle these colors…M  1: Like everything goes  2: A universal code…M  2: A color code that means When I see a product with 1 nothing else is good or less bad? …M  2: It is not so good…M  2: If nothing else has a red it is not so bad compared to the other you see green and you say maybe it is good ... M |  |
|  | **Graphic and language elements** | 1: The green color that tells me?  2: What's good ...M  2: Recommended ... M | 1:They have a percentage, they are 3 tells you sodium fats, it is a super healthy product, but you see that it is red, orange and green above, this is intermediate 1: Intuitive worked? One more step is to show them as if they were in the store on the shelf, they arrive, they stand on the shelf and they see all the products that seem to them and as they see them, tell me?   2: Immediately you go to the healthy ones, the most green bars and an orange, the one that is 2 red and one green stands out ... B |  |  |  |
|  | **Utility** |  | 1: What makes you healthier than the other?  2: The 2 green colors ... M |  |  |  |
| ***1, Moderator; 2, Participant; M, Medium Socioeconomic Level; B, Low Socioeconomic Level*** | | | | | | |

**Health Star Rating *(HSR)***

| **CODES** | | **Adolescents between 13 and 14 years old** | **Young adults between 21 and 23 years old** | **Mothers with children between 3 and 12 years old** | **Parents with children between 3 and 12 years old** | **Seniors between 55 and 70 years old** |
| --- | --- | --- | --- | --- | --- | --- |
| **Acceptability** | **Taste** | 2: I see it and it doesn't attract me at all…M  2: It doesn't appeal to me, but I like the color… M 2: It's confusing…M  2: Yes…M  2: It doesn't get my attention, it confuses me… M |  | 2: You know if he calls me because of the colors and shapes, but he doesn't say anything to me, if he calls me but then what? ... M 1: How do they see it?  2: It looks better…M  2: It looks great…M   2: For the color, it helps a lot ... M |  |  |
|  | **Attractive** | 2: for example, that says 3.5 index of healthy stars, that is that it is less healthy than that…B  2: For the stars…B  2: and for the colored ... M 2: It is ugly…B  2: It is not understood so much…B  1: Do not you understand?  2: Not so much… M 2: it doesn't stand out its color I can't see it from here… M 1: Orange doesn't attract much attention. Give me suggestion of colors.  2: Green…M  2: Blue…M  2: Yellow…M  2: I would say that the stars would love and everything else all black ... M | 2: You see more certificate…M  1: Do you see more a quality index or more a health index?  2: Quality ... M 2: Super easy…M  2: More healthier stars…M  2: 5 stars healthier ... B 1: CO:  2: Super easy.  2: More healthier stars… B 2: There's a meter, like when you measure speed, light water, it's understandable…B  2: You don't know how much sodium to eat, how many fats, but that's telling you a percentage, already qualified, to know if it's good or not, you don't worry anymore ... B | 2: I get noticed because it looks like an athlete…M  2: It's like medals ... M 2: I don't understand…M 2: They are stars…M  2: They are like movies and movies…M  2: By category…M  2: If you have 5 stars ... B 2: 5 Very good…B  2: Healthy…B  2: A good product…B  2: Reliable ... B 2: Confused, I understood that the use of stars is healthy 3.5 is healthy, 5 is healthier ... B | 1: CO if I understand that they are telling me:  2: It's like the evaluations you give on Facebook…M  2: On Netflix…M  2: To hotels 2: I like it because it doesn't force you to buy the healthiest thing that sometimes is the most expensive , you can go for the 2nd best option…M  2: And it is well understood but unlike the other you have no one to certify it ... B 2:That seems better to me…B  2: It seems to me still a seal of quality is very similar to Pigrims used as a seal of quality was very similar…B  2: The message is clearer tells you this is healthier…B  2: That is very confusing the message, the colors that have this ... B | 1: Is this the proposal as you see it?  2: It already encompasses everything…B  2: Gives the range ... B 2: It's for stars…B  2: As a 5-star hotel…B  2: It's the same as the one with numbers, the highest 5 ... M 2: It's like the speedometer, there it is he stopped and you give him 3.5…M  2: Ok he lacks information…M  2: In that he is tall or short ... M |
|  | **Cognitive Charge Perceived** | 2: The colors are not calling me…M  2: At first glance I do not pay attention to what they say ... M |  | 1: What part does not convince me?  2: At first glance I didn't understand much…M  2: My mom would understand that more than that…M  2: I like having 5 stars is the maximum ... M 1: And if I had 0 stars 0 1 star is it?  2: It is not reliable ... B 2: It is fine because on the tablets, many of us do not understand, what is sodium, that marks that I go through a test, children when they study they put a star, whoever has 5 is excellent quality ... B |  | 1: This more or less the color would change for something more flourishing, highlight at sight  2: Different colors, numbers…B  2: Contrasting colors…B  2: Marked the bad red, the green minus…M  2: Do not occupy the orange, only yellow green red…M  2: It depends on what you want to spend what you buy ... M |
| **Objective understanding** | **Design** |  | 1: 1 for being the average, is it in which world moves healthy or negative?  2: In the middle…M  2: In the middle…M  2: This can tell you the 2 things…M  2: It's in the middle…M  1: Is a star pipe healthy or not?  2: No…B  2: No…B  2: Unhealthy…B  1: Zero stars?  2: Nothing healthy ... B |  |  |  |
|  | **Central Message** | 1: What are you trying to tell them? I see it on a label and what is it about?  2: I don't understand…  1: No.  2: No  2: Where it says how healthy  2: The rating… M 1: Healthy stars. If I have 5 stars I have a product  2: Very healthy  1: If I have 3.5  2: Medium  2: Medium healthy  1: 1.5  2: Under  1: 0  2: Nothing healthy ... B 1: Let me show you how the product looks physically To give everyone the same chance to evaluate everyone else, for example, I have a box of cereal. Have you seen it yet? There it has its seal Yes? What does it say? How many stars?  2: 1.5…B  1: What are you telling me?  2: It's not very healthy…B  2: Not so healthy ... M 2: The presentation is fine, but there is a lack of information…M  1: Lack of presentation, apoco How do I expect to see it, I hope to see all the columns in the whole product or just one strip?  2: Only one strip...M | 1: The more stars:  2: Healthier…B 2: The healthiest are those with 5 stars…M  2: Better ... M | 1: What you are doing is rating the level of health of a product, it has 5 stars is very healthy, 3.5 is more or less Do you have 1?  2: It's not healthy ... B 1: What are you saying compared to this side:  2: It's less healthy…B  1: Why?  2: They have 3.5 stars in qualification…B  2: Therefore we are not educated, and the product is more balanced…B  1: And here as I see it I understand if I see a product with this seal and this with another seal?  2: That's better…M  1: The one with 5?  2: Yes…M  2: It is healthy, the 5 is healthy ... M 1: What if it had 0 stars 0 1 star is it?  2: Not reliable…B  2: Low quality…B  2: I would think about buying  2: 3.5…B  2: Regular ... B | 1: Where is it located on the scale?  2: In the middle…B  2: In the middle throwing healthy  1: In the middle throwing healthy  2: More good than bad...B 2: 5 is good…B  1: 3 and 5 what will it be?  2: About…M  2: There is driving you in the area of ​​the good 3.5 is not so good, but still good…M  1: And the other was driving  2: In the negative…M  2: And there are less than 3.5 and what put or nothing else 5 and 3.5…M  1: It is not a third or fourth possibility account, if I had half a star  2: .5 ... M 1:CO (label) if I understand that they are telling me:  2: It's like the assessments you give on Facebook…B  2: On Netflix…b  2: To hotels ... B | 1: If I have 5 stars, is it good?  2: Yes  1: 3.5  2: Medium…B  1: Half star  2: Here 5 stars is very expensive, very healthy and if the stars go down it is very cheap…M  2: Not healthy or the product is good for the stars… M |
|  | **Graphic and language elements** | 1: Let's see why we doubt it? Where is the possibly complicated?  2: How far the color goes or so.  1: This shadowed.  2: Yes  1: What else? 5 is healthy, why is 5 healthy?  2: Because 5 in healthy ... B |  |  |  |  |
|  | **Utility** |  |  |  | 1: And here?  2: And here it is telling you that it can't hurt you…B  2: It tells you healthy…M  2: The legend below says healthier and then the more stars it is healthier… B |  |
| ***1, Moderator; 2, Participant; M, Medium Socioeconomic Level; B, Low Socioeconomic Level*** | | | | | | |

***Healthy Choices***

| **CODES** | | **Adolescents between 13 and 14 years old** | **Young adults between 21 and 23 years old** | **Mothers with children between 3 and 12 years old** | **Parents with children between 3 and 12 years old** | **Seniors between 55 and 70 years old** |
| --- | --- | --- | --- | --- | --- | --- |
| **Acceptability** | **Taste** |  | 2: It's from anonymous alcoholics…B  2: From seafood restaurant…B  2: It's nice but it doesn't tell me anything, although it says it's healthy, but it doesn't tell you the benefit…B  2: It exists in new products… B | 2. I like it…M  2. I liked it…M  2. No, it seems that it is healthy but it does not say that it contains, because it is healthy…B  2. It is like the stars…B  2. It is the approval…B  2. They already approved it is good… M | 2: The product is healthy…B  2: Reliable…B 2: From SAGARPA ... B |  |
|  | **Attractive** | 1: What else does this scroll mean?  2: What is intake? …B  2: Eating…B  2: What do you eat…B  2: Digestion…B  2: The word is derived intake 2: It is well directed…B  2: What is recommended by experts… B | 1: What does the message say?  2: Based on international recommendations, several countries are authorizing it, they recommend it…M  1: Does that beat me?  2: Yes…B  2: It is reliable…B 2:The bad thing is that, if there are no good products, you don't know which ones are bad... B | 2: You are specifying…B  2: Pigeon…B  2: It says that it is based on international intake recommendations there is already someone…M  2: Someone who is supporting you that is reviewing the products ... B |  | 1: And how they see that because in others they told me you are healthy, you are more or less and this is healthy, no, how do they see it? It is very sharp or good  2: If it is good…B  2: If healthier…B  2: More reliable…B  2: If it is the same product it can be a pigeon, not a somewhat red dove, as you would distinguish a popcorn than if another does not ... B |
|  | **Cognitive Charge Perceived** | 2: What is recommended in international intake recommendations ... (laughs) …M  1: What does that mean? How do you understand it? How do you understand it with your words?  2: Mmm, no, I don't know .... M 2: But maybe they're lying to you, it's not that healthy and they already bring it, they just put you ... B |  | 1. Would they wear it?  2. To healthy products…M  2. That logo is very decisive…M  2. It doesn't have it you don't know…M  2. It drifts you… M | 1: What could you tell me about this label? 2: It would give me the guarantee that in this case it is healthy (product) ... but anyway I would have to do my job of making my numbers to know how many calories I need ... M | 1:And then these little letters we can see them?  2: Based on recommendations…M  1: Based on international intake recommendations  2: You can eat it with confidence…M. 1: As I feel like a consumer, I feel good, they are informing me how do I feel?  2: I feel good because I am certified ... B |
| **Objective understanding** | **Design** |  |  |  |  |  |
|  | **Central Message** | 1: Tell me what you say, what you communicate?  2: That 100% healthy, not so 100%, but there is one ... B 2: But maybe they are lying to you is not so healthy and they already bring it, they just put you ...B  2: That's why they bring the nopal tortillas, the tostadas “Sanísimo” and those things bring them ps and apart they bring one of those too ... B | 1: What does the seal you want to tell me mean?  2: How is it right…M  2: It's a popcorn…M  2: Popcorn means approved…M | 1. The logo you want to tell me, the elements you communicate, the popcorn that tells me?  2. It's good…M  2. It's down…M  2. It gives confidence ... M 1: How do you see that make you doubt?  2: Now that they put the stars, we chose 5 to healthy nobody chose 3.5, we went to healthy and this would be the same…B  2: Your child likes a product and does not have the healthy seal…B  2: I do not buy it, surely there is another one that has it ... B | 1: What are you communicating to me? 2: They force me to be selective, to choose better ... M  2: The product is healthy…B  2: Reliable ... B | 1: How do I differentiate the one that is healthy and the one that is not? With seal or not seal or with seal of taches and doves or how? Also international seal when it is negative:  2: Yes…B  2: To be able to consume it only like this and this gives me confidence…B  2: With seal and the one that does not seal ... B |
|  | **Graphic and language elements** | 1: How could I improve it?  2: If the label says it's a healthy option, say why reasons is a healthy option… M |  | 2: It doesn't say anything ... it's a healthy option, but it doesn't tell me how healthy it is to decide if I buy it ... B |  |  |
|  | **Utility** | 1: Everyone had. What I want to ask them is, if you take this one, what else would they take? This this.  2: The seal ...M  2: Seal ...M  2: I don't care ...M  1: You don't care, exactly the same 1: I wouldn't care, 7 if they would go for the seal, what I wanted to say, those who went for the seal, What would you think have no stamp?  2: Which is healthier…M  1: Healthier or less the one without a seal?  2: The one with the seal is healthier ...M 2: The one with the seal is more  recommended ... M 1: Let's see this question and the healthy option?  2: Well, they don't bring it ...B  2: The healthy ones just bring it ... B |  | 1. Does it help to choose?  2. Yes…M  1. This father is healthy and unhealthy if or that of scale  2. That of scale…M  2. This is more expensive for being healthy, a person who does not have the possibility… M | Someone lacked figures or that's fine  2: It's okay…M  2: It's a seal of quality ... M |  |
| ***1, Moderator; 2, Participant; M, Medium Socioeconomic Level; B, Low Socioeconomic Level*** | | | | | | |

***Warning label-Red***

| **CODES** | | **Adolescents between 13 and 14 years old** | **Young adults between 21 and 23 years old** | **Mothers with children between 3 and 12 years old** | **Parents with children between 3 and 12 years old** | **Seniors between 55 and 70 years old** |
| --- | --- | --- | --- | --- | --- | --- |
| **Acceptability** | **Taste** | 2: Okay, the colors are flashy...M |  |  | 2: The colors are very aggressive, because normally that type of shape is in the curves…B  2: Signaling of cars ... B | 1: Let's see how it looks in red?  2: Yes…M  2: Better…M  2: Much better… M |
|  | **Attractive** | 2: It prevents you more ... M 2: Not if it looks red as if it is a precaution, it is alerting you to something ... M 1: Which one tells you more danger red or black?  2: The red ... M 1: The black tone is more visible. Let's see if we already know that in our mind red is equal to danger, in our mind black is equal to:  2: Death ... M 1:is BN-R, ready turn it over and there you will see what do you think ? Tell me  2: It looks boring ... B 1: How do I expect it to appear on the label?  2: Same as the other…B  1: The more seals the more bad. And the less seals less bad, it is the same.  2: Yes. ... B 2: It has no striking color ... B 1:See how it looks on the shelf? Tell me and compare me? How are you?  2: It looks ugly…B  1: Why?   2: It looks boring. ... B | 1: What are you wanting to communicate to me?   2: It is dangerous…B  2: Do not buy it…B  1: It is dangerous do not buy it  2: Caution…B  2: It is dangerous, it tells you high, high…B  2: It is a warning symbol…B  2: Do not have that would be good…B  2: It is the idea in products chemical, it comes like this you shouldn't take it ... B 2: In the middle…M  2: Healthier…M  2: It's because of the colors, red you see it badly, green gives you more confidence, says negative things, the other one said positive thing so you could welcome…M  2: It is positive but the square is equal to the other ... | 1: With the color that changes?  2: In school they are handled…B  2: As strong, it is bad, much worse still…B  2: If I imagine it not so much…B  2: If I like it... B 1: How would you say this color is?  2: Very striking…B  2: It's the same for a while, I just wanted to change the color and see if we stay with the other…B  2: We saw a prettier one ... B 2: It looks pretty…B  2: It looks better ... B 2: I didn't think it was good because in the questionnaire they gave us this, it looks black, and being black doesn't attract attention…M  1: I didn't call the attraction, it wasn't attractive  2: They seem like traffic signs ...M 1: Let's see here I change them in red?  2: old…M  1: Better…M  2: This more striking…M  1: Red as color, what does it convey to me?  2: Alert…M  2: Caution…M  2: Prevention ... M | 2: It is very strong in sight ... M Caution  2: Exactly  2: A danger  2: I say it's okay is a water warning  2: Okay, but I liked the other one more.  2: I feel very aggressive  2: If it is very aggressive  2: And less compressive ... M  1: For the color.  2: Yes, because of the color… B 1: It doesn't attract attention, but what does it mean?  2: I think he wants to make me understand that the product has a certain risk  1: There is a certain risk  2: It is an alert message  1: A letter message  2: Stop, observe, read, read how much sugar has , how much sodium.  2: That can have an impact on your body so to speak .... B 2: We will not buy anything.  1: That is the feeling that leaves you, I am not going to buy anything anymore  2: What happens is all the same in the All Brain, which in theory is minus  2: It has 2 reds.  2: At first glance, I don't like it, they seem like quality stamps ... B 2: It doesn't attract attention  2: You don't buy the product if you see it in red  2: It's aggressive  2: The figure doesn't I like them, they look like honeycombs ... B 2: It is that there in red attracts attention ... M | BN-R ready, what do you think?  2: That if I like  1: IS the one we had in black and white  2  : Is it better in red 1: Is it better in red?  2: Yes  2: It's much better  1: Why tell me? It is more attractive, what more achievement?  2: Call more attraction  2: It is a precaution  2: Excerpt  2: It says loud look at me   2: It looks harmoniously the black saw it as  2: Very sad  2: Very opaque  2: Off ... M let's see the first BN-R comes out Tell me how you see it this?  2: Good  2: I like it more so black  1: Why?  2: It looks better  2: It's not all in black  2: More than anything because of the high ... B 2: You get to see the letters better as the red stands out more white  2: Call more attention  1: If you say red is high, I am used to  2: To see the red that is high  1: What else can you tell me about this?  2: A stop to consume them ... B |
|  | **Cognitive Charge Perceived** | this? It can make you a year or if you eat something it can take you to the calaca, what is more powerful as a message?  2: The calaca ... M |  | 1: By this you mean a little undress the products.  2: Yes  1: Evidence them.  2: If the message is super clear ... M |  |  |
| **Objective understanding** | **Design** |  |  |  | 1: Which one would you buy? which is less harmful?  2: I think the problem is the aggregates as you say and the All Brain has something else…M  2: I would buy the Torstas, which at first glance it looks like it doesn't have…B  1: They decide more for the photo of the product than by the seals.  2: Yes…B  1: If it were for stamps which product there you say is the one that should suit me for pure stamps?  2: the one on the bottom right has no stamps .... B |  |
|  | **Central Message** | 1: How do I expect it to appear on the label?  2: Same as the other…M  1: The more seals the more bad. And the less seals less bad, it is the same.  2: Yes…M | 1: The question you asked with the other here as it would appear, a rhombus, several?  2: There may be several, this high in 2 things, having one already influences that it has an excessive issue, now that it has 2 you no longer buy it, it has the 3  2; It would be accumulating studs ... B | 1: What is the message, what are you wanting to tell me?  2: What contains negative ... M | 1: Leave it ambiguous  2: Is that the information…B 2: I would go for the products that have a seal…B  2: As you need other knowledge as more to understand what is energy? …B  1: Compared to the ones I currently have, is this better than that? ... B 1: And if I have 1, what do I think?  2: It's not that high…M 2: Seeing them all the same color and shape, I don't see it anymore, everything is bad…M  2: It's not that high…M  2: Not what would happen you wouldn't understand him well ... M |  |
|  | **Graphic and language elements** |  |  | 1: Red as color What does it transmit to me?  2: Alert…M  2: Passion ... M | 1: The red color, besides being a warning, what else do they tell me, toxic danger?  2: Stop…B 2: See the number of stamps, and then you see what each stamp says...B | 2: Red gives you life…M  2: It catches your attention, but it still doesn't tell me what is good about it? You are limiting me. It's like seeing me I have this and that bad ... M 2:I stop more to see why it has that logo... B 2: That would be fantastic if they did it…B  1: Why would it be fantastic?  2: We would not waste time…B 2: Stop to consume it, so they are in red  2: It would not cost work to choose between this and the other…B  1: I do not waste time what else?  2: And you are no longer reading everything nutritious on the back, I would just look at the stamps... B |
|  | **Utility** | 1: What you have higher will appear Do you see it easy to understand, of course?  2: If it is easy to understand, but it gives little reading ...M |  |  | 2: Red gives you life…M  2: It catches your attention, but it still doesn't tell me what is good about it? You are limiting me. It's like seeing me I have this and that bad ... M 2: I stop more to see why it has that logo ... B 2: That would be fantastic if they did it…B  1: Why would it be fantastic?  2: We would not waste time…B  2: It would not cost work to choose between this and the other…B  1: I do not waste time what else?  2: And you are no longer reading everything nutritious on the back, I would just look at the stamps ... B |  |
| ***1, Moderator; 2, Participant; M, Medium Socioeconomic Level; B, Low Socioeconomic Level*** | | | | | | |

***Warning label-Black***

| **CODES** | | **Adolescents between 13 and 14 years old** | **Young adults between 21 and 23 years old** | **Mothers with children between 3 and 12 years old** | **Parents with children between 3 and 12 years old** | **Seniors between 55 and 70 years old** |
| --- | --- | --- | --- | --- | --- | --- |
| **Acceptability** | **Taste** | 1: Since it has a lot of sugar a lot of energy, it's like drinking coffee ... M 1: For example, let's see?  2: High in energy, when I'm in the morning, I need energy ... B  Tell me everything you want to tell me about this BN(label)?  2: She's ugly ...B  2: Very simple ...B  2: She doesn't have the percentage of each one ...B  1: She doesn't say the percentages she just says loud ...  2: Very boring ...B  2: Dark and soon ...B  2: Sad…B  2: Racist .. B 1: What else?  2: No, no joke ...B  2: I didn't like it ... B | : Whatever as a transit symbol to which it looks like?  2: Stop…M  2: Stop…M  2: The idea is good but…M   2: Show us the products this is high in sugar or fat ... M | 2:I don´t like the black color…M  1: Black not because?  2: She looks sad ... M 1: Is this figure where I see her?  2: In signs…B   1: Is it for what?  2: High…B  2: Alert… B | 1: Is the message here?  2: You are going to die  2: High in calories and you are going to die, high in sodium if you eat it you are going to die, high in sugar you are going to die and nothing more… M  2: It is very complicated if they put these labels, do not buy this “cannon”, with high sugars, sodium ... M 1: It seems those stop  2: Transit…B  2: But if it catches your attention by the high ...B |  |
|  | **Attractive** | 2: Well, by the parable of this, it is understood that it is above what is necessary to put it this way… M 1: For example, let's see?…B  2: High in energy, when I am in the morning, I need energy ... B 1:do I understand him or not?  2: If you understand him a little…B  2: He is understood very quickly there he says high in energy and if he would not say no…M  2: This is the figure of the stop…M  1: Is the figure of the stop what does that tell you?  2: Stop…B  2: Look…B  2: It has no joke ... B 1:This is how the products would look on the shelf ...  2: It looks pretty ...B  2: It looks sad ...B  2: It ruins the presentation…B  2: The color ... B 2: It is anticipating something…M  1: It is protecting us from something What?  2: Of the bad feeding…M  2: Of the characteristics of the product ...M 1: The majority takes the red one Tell me why?  2: Because it attracts more attention ...M | 2: Everything is high, without numbers…M  2: Very off…M  2: Very ´X´…M  2: Dangerous ... M  2: Ugly color…B  2: Not understandable…B  2: It goes unnoticed…B  2: Bored as usual ... B 2: Roads…B  2: It's the stop… M 2: I don't like this father with a black color, better at a traffic light, but like this…M  2: If you don't like attention, you lose ... M | 2. It depends on what the product has…M  2. It depends on each product…M  2. The color does not look pretty…M  2. It is not flashy ... M 2. It depends on what the product has…M  2. It depends on each product…M  2. It does not look nice color…M  2. Not flashy ... M 1. Black is not very father?  2. It's sober, but more dramatic…M  2. You see it with greater impact…M  2. It has more impact than black…M  2. Let's see what it has because black ... M 2: It's understandable…B  2: Black is bad…B  2: That's why All that is bad…B  2: All that is high…B  2: The skull is missing in the middle…B  2: So that to put several specifications, in the end everything is bad, with a black label and you specify what is wrong…B  2: I do not like it puts the bad and does not explain the good of the product...B | 1: BN:  2: It is clear but I want colors…M  2: Very alarming…M  2: It is very aggressive for the consumer that is fine but how tall is it? It doesn't leave me, stop if ... M. 2: Nutritional table of a funeral…M  2: Of an additive ... M 2: It looks father…M  1: In some nuts  2: It looks pretty…M  2: Pro look at that sign I see it for very natural products 100% natural…M  2: Coca will not be…B  2: It would look like an apple…M  2: It can't be in junk food ... M 1: What else are you telling me?  2: Tall, tall, tall how I'm going to consume that product if everything tells me loud…B  2: It looks very funeral, all black…B  2: It's going to die ... B | 1: How do they see it?  2: The color  2: The color if  2: The red  2: Everything is high ... B 2: It attracts attention and gives me a reality, it is very explicit ... B |
|  | **Cognitive Charge Perceived** | 2: That is not healthy.  1: Why do you say it is unhealthy?  2: Because it says high in sodium and energy and all those things, this product is not leveled for the health center ... M 1: It is understandable almost ¾ and I like it, what do we do to please me? What could he have or what he lacked?  2: Well, knowing what it is that is high and so ... M 1: Let's see how it looks physically in the products, do you think? See, in real physical products. They look like this, for example I go to Sam's and I would buy these that would look like this.  2: This father the image ...  2: It does not attract attention ...  2: Cover the image ...  2: How it hinders ... B If I see this I would know which one to buy or which not to buy?  2: Yes ...  1: For example, which ones would you take?  2: The one with 1 ...  2: It depends on what you want to take care of too ... B 1: One thing is that it doesn't look pretty and another thing that doesn't attract attention, for example, my 200 kg grandmother at the peripheral in balls doesn't It looks pretty, but it's going to get attention, I'm sure, isn't it going to get attention or isn't it going to look pretty?  2: It does not attract attention  2: It does not attract attention  1: It does not attract attention I will not see it?  2: You see it, but it doesn't look good ... M 2: It attracts attention for the same and the color so dark, as if it prevents you from something ... M | 1: If you think so, we saw it like this and now we see the physical product and then as if we were in the store it seems to them. We already saw it that way now I want to teach product ideas. As it would look, the product would be something like that, it is empty, but imagine it is full and to see this stamp on these products, how do you see it? This is the red cap. What happened?  2: First it takes up a lot of space and takes away the image  2: Evade the theme ... M | 1: We see the following label, how do you see it?  2: It means you have more things that are not good ... M |  |  |
| **Objective understanding** | **Design** | 1: The red ones for?  2: They lick my attention more, black seals are much bigger and you distinguish them more ... M 1: Have you never seen black, I don't believe them?  2: We don't know what it means  2: I've obviously seen it but I don't know what it means if they put it on a product  1: But where have you seen it? …B  2: The color, but not so…B  1: But where have you seen the color black?  2: In some signs I think ... B 1: Is the figure of the stop, what does that tell you? …B  2: Stop…B  2: Look ... B | 1: A precaution, the color I'm sorry, what feeling does it cause me?  2: Sadness…M  2: Negativity ... M |  | 1: Which one would you buy is less harmful than these  2: I think the problem is the aggregates as you say and the All Brain has something else…M  2: I would buy the Torstas, which at first glance it looks like it doesn't have…M  1: SE they go more for the photo of the product than by the seals.  2: Yes…B  1: If it were for stamps which product there you say is the one that should suit me for pure stamps?  2: the one on the bottom right has no stamps ... B | 2: Yes  2: I like the idea you have, we all specify the idea of ​​stop even if they put it in red I would call much more…M  2: In red yes ... M |
|  | **Central Message** | 1: Would you take which of the 3?   2: Red…M  1: Why red?  2: You see the black one, it is organic, you should know simple, I take the red ones that should know better ... M 1: That is, nothing else is the anger with children? There is the difficulty, from what year could they understand it?  2: Of the 8 ... B 1: Why do you say it is unhealthy?  2: Because it says high in sodium and energy and all those things, this product is not level for the health center ...M  1: And why does one have 3 and the other has 2 tell me?  2: Well, because one only has more than another ... M 1: What do I understand with this label?  2: What is high in everything…B  1: And when something is high in everything?  2: It's bad for your health…B  2: We don't buy it…B  2: It depends on how you interpret your stop ... B |  | 1: What is labeling telling me, is it healthy or healthy?  2: In the middle…B  2: It says high in sodium ... B | 2: Leave it ambiguous…B  2: Is that information…B  1: As you need other knowledge as more to understand what is energy?  2: Yes…B 2: Yes ... B 1: Let's see one has 2 and the other has 3 high, what do you mean?  2: It has higher…M 2: It is more bad ... M | 1: Very quickly I would like to be told What is the purpose of this or what remains for me wanting to convey this type of stamp?  2: Don't buy…M  2: Caution…M  2: It's been that there have been a lot of cancer problems lately from diabetics, children who are diabetic so what they said about junk food has to be stopped, but we ate the junk food from children and it didn't hurt us , so something is being put to the product so that right now ... M 1:High in energy How do I interpret it? I'm so sorry? And is it healthy or not healthy?  2: If very healthy…M  2: Healthy ... M 1: Here you have 2 and here you have 3, what is that telling me?  2: That they have calories…B  2: They have more things ... B |
|  | **Graphic and language elements** |  | 1: High What is the household item that you want to tell me?  2: Like you don't buy it…M  1: Don't buy it, what else does he tell me with this stop?  2: As a precaution ... M |  |  |  |
|  | **Utility** | 1: What you have higher will appear Do you see it easy to understand, of course?  2: If it is easy to understand, but it gives you little reading ... B 2: Yes…B  1: What else can you tell me about this? Bored, do I understand you or not?  2: If you understand him a little…B  2: He is understood very quickly there he says high in energy and if he would not say no…B  2: Is the figure of the stop ... B | 1: And what effect would it have on my putting 2 stamps?  2: That is very unhealthy and they are bad, it would be good to put a good one and a bad one…M  1: A good one and a bad one  2: If high and low ... M 1: How would you be physically in the product?  2: It depends on what you bring, Coca brings sugars, sodium…M  1: We will have 4, 5, 3, 2 so it would be   2: If it depends on what I had…M  2: It's like the board…M  2: It's the same as the table ... M | 1. One had 2 other 3   2. It depends on what the product has…M  2. It depends on each product…M  2. The color does not look pretty…M  2. It is not flashy…M  1. By which stamps would it take me?  2. None…M  2. The one on the right, by big guys…M  2. I would not take any, because it is not high on something ... M 1: You can accumulate 5, 3, 2 faults, it is like accumulating fines how do you see?  2: They would no longer buy it…B  2: It is on top, there they take more…B  1: It would not be like that, it can have up to 5 or none  2: It is saying that the product is not very bad …B 2: It does not say what is wrong or good…B  2: Even if I have 1 it's bad ...B |  | 2: I do not know if I am getting ahead of the other posters but here I see that there are 5 elements to take into account and in the others there are only 3 from there I think they are starting badly that it is not all the information they have to give us and if they are handling colors, logos nothing more, but I continue with the fact that they are not telling us what the product contains and then it would be 5 by 3 15 logos that they would have to put high, medium and low as because they are saying I am hypertensive, I'm diabetic, there are allergies ... M |
| ***1, Moderator; 2, Participant; M, Medium Socioeconomic Level; B, Low Socioeconomic Level*** | | | | | | |

**5 Color Nutritional Labelling *(5-CNL)***

| **CODES** | | **Adolescents between 13 and 14 years old** | **Young adults between 21 and 23 years old** | **Mothers with children between 3 and 12 years old** | **Parents with children between 3 and 12 years old** | **Seniors between 55 and 70 years old** |
| --- | --- | --- | --- | --- | --- | --- |
| **Acceptability** | **Taste** | 1: What will make you more or less healthy not so healthy?  2: The letters and colors…M  1: Is it visible by itself?  2: More or hands, if it were bigger or the size...M 1:RO (label), don't answer yet let's see the products Let's see tell me? Ready as it looks?  2: I do not understand anything ...B  2: Me neither...B 1: Well, here we go, let's see, for example, here it would be. See?   2: It looks pretty...B  2: Single row... B | 2: This ugly…M  2: The periodic speech…M  2: It would be one for each product... M 2: It is not understood…B  1: I do not understand it  2: I would not know what it is ... B | 2. Looks like optics…M  2. Vitamins…M  2. It's an Abaco…M  2. A children's work of the molecules…M 1:What will it be telling me?  2. It is a traffic light…M  2. Do not buy red flat…M  2. The, purple, lilac, pink that is ... M | 2: What does A mean, vitamins…B  2: Abacus… B | 2: It doesn't tell me anything…B  2: Me neither does…B  2: You don't know what A, B and C means…B  2: It's from the product is sodium sugar… B |
|  | **Attractive** | 1: What is this scale telling you?  2: That the 1st letters are the best ...M  2: For the type of color if I would go, that until the end is red, before there is pink...M 2: The presentation is fine, but information is missing ...M  1: Missing presentation ,How do I expect to see it, I hope to see all the columns in the whole product or just one strip?  2: Just one strip ... M 1: The D of what would you tell me?  2: “Chafisima” or more or less “chafa”…M  1: The B that would tell me?  2: More than acceptable ... M 2: It's from vitamins ... B. 1: Here as long as the circle is bigger it indicates how healthy everything is ...  2: And what does E…B  2 mean: What is the vitamin for there ...  2: It's almost there same as the other one that I liked the VE ... B | 2:It's like going to a 5 star hotel or one… B | 1:What will it be indicating to me?  2. It is a traffic light…M  2. Do not buy red flat…M  2. The, purple, lilac, pink that is ... M 1:RO (label). What do you think?  2: No…B  2: I don't understand anything…B  2: It looks like a traffic light card…B  2: If I understood that it is A, B and C  2: It's like what it brings…B  2: Like at school ... B  2. The plane red doesn't buy…M  2. The, purple, lilac, pink that is ... M |  | 2: You get confused…M  2: You try to identify which one is bigger to know if it suits…M  2: You are in a hurry…M   2: Look at that one brings the alphabet ... M |
|  | **Cognitive Charge Perceived** | 1:to fill in the last label I would like us to see the letter RO, yes? Are you ready?  2: No yes, I don't understand anything about it ...M  2: Does it have to do with vitamins or something? …M 2: This one is healthier A, less healthy E… M | Who does he know about?  1: It's already clear that I don't.  2: Vitamins…B  2: Vitamins can be What else? …B  2: It looks like a set of cereal boxes…B  2: It could be talking about very high vitamins, vitamins A, in vitamin B…B  1: What other interpretation is worth? ... Any other explanation? Meaning besides vitamins, A, B, C  2: It can be on a small board, the first one is saturated fat, the second one is other fat, the third one is sugar, the fourth one is low in sodium…B  1: Let each one of these be worth every one of these here ... B | 2: A and C that means calories…B  2: As in school…B  2: I didn't understand…B  2: The A what it means? …B  2: Good…B  2: High in fat…B  2: Low…B  2: Cholesterol… B |  | 2: It has the smallest quantity of a product, there is a larger A, it is the product that has…B  2: The one with the smallest ones, the largest has more…B  1: Less or more what?  2: Calories …B  2: Sugars…B  2: Sodium… B |
| **Objective understanding** | **Design** |  |  |  |  |  |
|  | **Central Message** | 1: If it is very healthy that strip I hope to see?  2: A…M  2: A…M  1: Is it acceptable?  2: C…M  2: C…M  1: And if it's Chafísima?  2: E…M  2: E…M  1: The D of what would you tell me?  2: Chafisima or more or less chafa…M  1: The B that would tell me?  2: More than acceptable ... M | 1: Nothing full or filled in pink or green And what does A, B, C mean?  2: For the color if it is good…M  2: Not much…M  2: Vitamins ...M 1: Any other explanation? Meaning besides vitamins, A, B, C  2: It can be on a small board, the first one is saturated fat, the second one is other fat, the third one is sugar, the fourth one is low in sodium ..B | 1. What do you want to tell me?  2. It has a lot of vitamin A or B and that is high in vitamin C…M  2. You have to know the nutritional colors well, so you can identify them…M  2. No…M  2. I don't relate it at all… M 2: If I understood that it is A, B and C  2: It's like what it brings…B  2: Like in school…B  2: A C that means calories ..B 1: In this example I just showed you we have 3 yogurt one in A another in B and one in C what is that telling me?  2: The C that can be calcium…B  2: Calorins...B | 2: What does A, vitamins means…b 2: I think that is an abacus… B | 1: Why is E here the biggest, here is B and here is C, what does it mean?  2: Vitamins…b  2: Nutritional value  2: It's confusing…b  2: It says nothing ... B 1: Here A is bigger and here B what is the best product?   2: In the alphabet A is the 1st…B  2: Having a natural appearance, that has extra ingredients…B  2: It has more calorie than light ... B 1: What could they mean?  2: Moderately healthy…M  2: Very healthy…M  2: Almost unhealthy…M  2: The information we are looking for is vitamin, it has a lot of vitamin C…M  2: Vitamin B…M  2: It has all the vitamins…M  2: They didn't put B12 ... M |
|  | **Graphic and language elements** |  |  |  |  |  |
|  | **Utility** |  |  |  | 1: Which one would go healthier?  2: A…B  1: The least:  2: E…B  1: And if it were more or less:  2: C…B  1: What is a, b, c  2: Vitamins… B | 1: What does health mean?  2: We would have to explain…M   2: By taking that out they are going to have to get information spots…M  2: A nutritional table…M  2: This complicated way of separating the garbage…M  2: We don't have that culture...M |
| ***1, Moderator; 2, Participant; M, Medium Socioeconomic Level; B, Low Socioeconomic Level*** | | | | | | |
