# Supplementary material for: Front-of-pack nutritional labels: Understanding by low- and middle-income Mexican consumers
Source: PLoS One. 2019 Nov 18;14(11):e0225268. doi: 10.1371/journal.pone.0225268 (PMC6860442; doi:10.1371/journal.pone.0225268)
Supplement: Attachment — Submitted filename: Rebuttal letter 1.docx [file pone.0225268.s009.docx]

**PONE-D-19-21167. Front-of-pack nutritional labels: Understanding by low- and middle-income Mexican consumers
PLOS ONE**

**Journal requirements/Editor’s comments**

1. When submitting your revision, we need you to address these additional requirements.

Please ensure that your manuscript meets PLOS ONE's style requirements, including those for file naming. The PLOS ONE style templates can be found at
[http://www.journals.plos.org/plosone/s/file?id=wjVg/PLOSOne_formatting_sample_main_body.pdf](https://eur01.safelinks.protection.outlook.com/?url=http%3A%2F%2Fwww.journals.plos.org%2Fplosone%2Fs%2Ffile%3Fid%3DwjVg%2FPLOSOne_formatting_sample_main_body.pdf&data=02%7C01%7C%7Cb23dcc7ca1344026be2f08d72c9948b0%7C84df9e7fe9f640afb435aaaaaaaaaaaa%7C1%7C0%7C637026909173892477&sdata=PiYhbum2VzT4FN%2FWVFh7ZHrU4KDFCrPfFtjpueHlu64%3D&reserved=0) and [http://www.journals.plos.org/plosone/s/file?id=ba62/PLOSOne_formatting_sample_title_authors_affiliations.pdf](https://eur01.safelinks.protection.outlook.com/?url=http%3A%2F%2Fwww.journals.plos.org%2Fplosone%2Fs%2Ffile%3Fid%3Dba62%2FPLOSOne_formatting_sample_title_authors_affiliations.pdf&data=02%7C01%7C%7Cb23dcc7ca1344026be2f08d72c9948b0%7C84df9e7fe9f640afb435aaaaaaaaaaaa%7C1%7C0%7C637026909173902488&sdata=tepr621I%2F1C8%2FxLlev1H1xWu0nkfUZ4c5dTReVS8L4g%3D&reserved=0)

*Response: We have made sure the revised version meets PLOS ONE’s style requirements.*

2. Please include additional information regarding the survey or questionnaire used in the study and ensure that you have provided sufficient details that others could replicate the analyses. For instance, if you developed a questionnaire as part of this study and it is not under a copyright more restrictive than CC-BY, please include a copy, in both the original language and English, as Supporting Information.

*Response: We have now included the demographics survey (S3 File) and the topics guide used in the focus groups (S2 File), in both Spanish and English, as supplementary materials.*

3. Please provide additional details regarding participant consent. In the ethics statement in the Methods and online submission information, please ensure that you have specified (1) whether consent was informed and (2) what type you obtained (for instance, written or verbal). If your study included minors, state whether you obtained consent from parents or guardians.

*Response: In the revised version we have included a statement mentioning that informed written consent was obtained before data collection for all adults. For minors, we mention that both, written consent from parents and written assent from minors, were obtained (Line 159).*

4. When reporting the results of qualitative research, we suggest consulting the COREQ guidelines: [http://intqhc.oxfordjournals.org/content/19/6/349](https://eur01.safelinks.protection.outlook.com/?url=http%3A%2F%2Fintqhc.oxfordjournals.org%2Fcontent%2F19%2F6%2F349&data=02%7C01%7C%7Cb23dcc7ca1344026be2f08d72c9948b0%7C84df9e7fe9f640afb435aaaaaaaaaaaa%7C1%7C0%7C637026909173912499&sdata=WKHFuFUkvI%2BhhWZsiMYP9Duw2%2BJk7GDxukBSvn8Hajw%3D&reserved=0). In this case, please consider including more information on the moderator (training and characteristics), and whether risk of bias was assessed.

*Response: The COREQ checklist was considered and we now include additional information thougout the manuscript to comply with the guidelines (Lines 132, 164). Additionally, we have included more details on the training and characteristics of the moderator (line 156). We have also mentioned that, in order to reduce a bias in responses from participants, the order in which the labels were presented was randomly assigned (line 186). Also, during the analysis we ensured a continuos dialogue between researchers to maintain consistency of the coding (Morse et a.l 2015) and mitigate interpretative bias.*

5. We note hat Figure S1 in your submission contain copyrighted images. All PLOS content is published under the Creative Commons Attribution License (CC BY 4.0), which means that the manuscript, images, and Supporting Information files will be freely available online, and any third party is permitted to access, download, copy, distribute, and use these materials in any way, even commercially, with proper attribution. For more information, see our copyright guidelines:[http://journals.plos.org/plosone/s/licenses-and-copyright](https://eur01.safelinks.protection.outlook.com/?url=http%3A%2F%2Fjournals.plos.org%2Fplosone%2Fs%2Flicenses-and-copyright&data=02%7C01%7C%7Cb23dcc7ca1344026be2f08d72c9948b0%7C84df9e7fe9f640afb435aaaaaaaaaaaa%7C1%7C0%7C637026909173922503&sdata=zxUg0xeTBT3flOSKF%2Fkxtiz1bD2tJ%2BMapoBEGY4xt3U%3D&reserved=0).
 
We require you to either (1) present written permission from the copyright holder to publish these figures specifically under the CC BY 4.0 license, or (2) remove the figures from your submission:

1. You may seek permission from the original copyright holder of Figure S1 to publish the content specifically under the CC BY 4.0 license.

We recommend that you contact the original copyright holder with the Content Permission Form ([http://journals.plos.org/plosone/s/file?id=7c09/content-permission-form.pdf](https://eur01.safelinks.protection.outlook.com/?url=http%3A%2F%2Fjournals.plos.org%2Fplosone%2Fs%2Ffile%3Fid%3D7c09%2Fcontent-permission-form.pdf&data=02%7C01%7C%7Cb23dcc7ca1344026be2f08d72c9948b0%7C84df9e7fe9f640afb435aaaaaaaaaaaa%7C1%7C0%7C637026909173932508&sdata=zp%2BsdqeSPT6%2Bd%2FmBiyCcc0h2pvrIPm%2FVCUHodxE0jsU%3D&reserved=0)) and the following text:
“I request permission for the open-access journal PLOS ONE to publish XXX under the Creative Commons Attribution License (CCAL) CC BY 4.0 ([http://creativecommons.org/licenses/by/4.0/](https://eur01.safelinks.protection.outlook.com/?url=http%3A%2F%2Fcreativecommons.org%2Flicenses%2Fby%2F4.0%2F&data=02%7C01%7C%7Cb23dcc7ca1344026be2f08d72c9948b0%7C84df9e7fe9f640afb435aaaaaaaaaaaa%7C1%7C0%7C637026909173942519&sdata=SE6EM8G3nXIXSyC%2B7ymm5b%2FitcoLL6rpPfyE3suxwIE%3D&reserved=0)). Please be aware that this license allows unrestricted use and distribution, even commercially, by third parties. Please reply and provide explicit written permission to publish XXX under a CC BY license and complete the attached form.”

Please upload the completed Content Permission Form or other proof of granted permissions as an "Other" file with your submission.

In the figure caption of the copyrighted figure, please include the following text: “Reprinted from [ref] under a CC BY license, with permission from [name of publisher], original copyright [original copyright year].”

2.    If you are unable to obtain permission from the original copyright holder to publish these figures under the CC BY 4.0 license or if the copyright holder’s requirements are incompatible with the CC BY 4.0 license, please either i) remove the figure or ii) supply a replacement figure that complies with the CC BY 4.0 license. Please check copyright information on all replacement figures and update the figure caption with source information. If applicable, please specify in the figure caption text when a figure is similar but not identical to the original image and is therefore for illustrative purposes only.

*RESPONSE: We have replaced the figure with a new one eliminating the brand of the products.*

6. We note that you have indicated that data from this study are available upon request. PLOS only allows data to be available upon request if there are legal or ethical restrictions on sharing data publicly. For information on unacceptable data access restrictions, please see [http://journals.plos.org/plosone/s/data-availability#loc-unacceptable-data-access-restrictions](https://eur01.safelinks.protection.outlook.com/?url=http%3A%2F%2Fjournals.plos.org%2Fplosone%2Fs%2Fdata-availability%23loc-unacceptable-data-access-restrictions&data=02%7C01%7C%7Cb23dcc7ca1344026be2f08d72c9948b0%7C84df9e7fe9f640afb435aaaaaaaaaaaa%7C1%7C0%7C637026909173952524&sdata=s3fcUsim%2Bu62RLjinNxrcXweRFwm1VpWBYiOcgNujVU%3D&reserved=0).

In your revised cover letter, please address the following prompts:

a) If there are ethical or legal restrictions on sharing a de-identified data set, please explain them in detail (e.g., data contain potentially identifying or sensitive patient information) and who has imposed them (e.g., an ethics committee). Please also provide contact information for a data access committee, ethics committee, or other institutional body to which data requests may be sent.

b) If there are no restrictions, please upload the minimal anonymized data set necessary to replicate your study findings as either Supporting Information files or to a stable, public repository and provide us with the relevant URLs, DOIs, or accession numbers. Please see [http://www.bmj.com/content/340/bmj.c181.long](https://eur01.safelinks.protection.outlook.com/?url=http%3A%2F%2Fwww.bmj.com%2Fcontent%2F340%2Fbmj.c181.long&data=02%7C01%7C%7Cb23dcc7ca1344026be2f08d72c9948b0%7C84df9e7fe9f640afb435aaaaaaaaaaaa%7C1%7C0%7C637026909173952524&sdata=hi2LP4n%2BZG551hHcbpWSFmxDAg4IG8SujPfpqsq2fGI%3D&reserved=0) for guidelines on how to de-identify and prepare clinical data for publication. For a list of acceptable repositories, please see [http://journals.plos.org/plosone/s/data-availability#loc-recommended-repositories](https://eur01.safelinks.protection.outlook.com/?url=http%3A%2F%2Fjournals.plos.org%2Fplosone%2Fs%2Fdata-availability%23loc-recommended-repositories&data=02%7C01%7C%7Cb23dcc7ca1344026be2f08d72c9948b0%7C84df9e7fe9f640afb435aaaaaaaaaaaa%7C1%7C0%7C637026909173962534&sdata=Druqk3pAqyONcpc2IN1n%2BjaMrOomrU4C%2Bwl5kIZ9eAU%3D&reserved=0).

We will update your Data Availability statement on your behalf to reflect the information you provide.

*Response: We uploaded o Figshare the anonymized transcriptions in Spanish, as well as the anonymized quatitative data set. Data are available here:* <https://figshare.com/s/7601afd0d47a1c52ab14>

*.*

**Reviewers' comments:**

**Reviewer #1:**

Review of “Front-of-pack nutritional labels: Understanding by low- and middle-income Mexican Consumers” for PLOS One

Summary
Our main objective was to explore the subjective understanding of labels that are currently used internationally among low- and middle -income Mexican consumers.
We performed two phases of 10 focus groups with adolescents (13-15 y), young adults (21-23 y), mothers of children 3-12 y, fathers of children 3-12 y and older adults (55-70 y). Seven FOPL were evaluated: Guideline Daily Amounts (GDA), Multiple Traffic Light (MTL), Chilean Warning labels (WL), WL in Red, 5-Color Nutrition Label (5-CNL), Health Star Rating (HSR), and Healthy Choice label. Data was analyzed with a triangulation of researchers using a content analysis, based on three codes: 1) awareness and use of the GDA, 2) acceptability, and 3) subjective understanding of labels

*We thank the reviewer for the summary of the manuscript.*

**Major comments**

Qualitative analyses with sufficiently large group (200 interviews) and appropriate for this type of analysis:

“Audios from focus groups were transcribed verbatim. The systematization and interpretation of the information was guided using the content analysis technique using Atlas.Ti version 11 [29,30]. We developed a priori central themes for our research questions before data was collected. Additionally, we also 218 developed a code catalog based on transcriptions. The analysis was based on three codes: 1) awareness and use of the GDA, 2) acceptability, and 3) subjective understanding of labels. Double entry conceptual matrices were developed for each code and participant group. Information was encoded and analyzed independently by the focus group moderator, as well as by two additional researchers (JVM and SPM), one of whom attended focus groups as listener.”

Timeliness of paper given the statement (lines 81-82): Thus, the regulation of FOPL in the country is currently being revised, to replace this system with one that better serves the purpose of decreasing sugar, salt and sodium intakes among the Mexican population.

Contribution: focus on lower income and education groups, which are the most representative of the Latin-American populations and generally the most nutritionally at-risk

200 participants (100 in each phase)

*Response: We thank the reviewer for the highlights of the manuscript.*

**Minor editing**

Data is plural-- change throughout ie Data was were analyzed with a triangulation of researchers using a content analysis, based on three codes

*Response: Corrected*

Line 65: insert which are before considered critical nutrients for chronic diseases

*Response: We included this information in the revised version.*

Line 92: nutritional quality of products, such as HSR and Nutriscore, may allow consumers to identify.

*Response: We have modified this sentence as follows: For example, the HSR and the Nutrisctore qualify the overall nutritional quality of a product, but they do not provide specific information on the content of critical nutrients such as fat, sugar or sodium.*

Line 103 In order to contribute to fill this gap in literature

*Response: We have now eliminated this text of the manuscript.*

Line 192: facilitator did not explain to participants the intended message of the labels

*Response: We have replaced this sentence by: The facilitator explored the subjective understanding of each FOPL without explaining participants the intended message of the labels.*

Line 235: foods (Table 1). Among those reading the GDA, 37% considered the label as incomprehensible or minimally understandable

*Response: We have changed this sentece as follows: Among those reporting to read the GDA, 37% considered the label as nothing or a little understandable….*

Line 309: They considered that it had too many confusing elements that made it difficult to interpret, and consequently they were not able to identify if the products were healthy.
*Response: We have rewritten this sentence to improve clarity*

Health Star Rating—this section is confusing –rewrite

*Response: We rewrote this section to improve clarity*

Line 395: Mexican consumers. These FOPL have been little assessed all together in previous studies. Rewrite as Few previous studies have assessed these FOPL together.

*Response: Corrected*

**Reviewer #2:**

Overall, I like this manuscript. The research is on an important topic: getting low and middle SES Latin American individuals to make healthier food selections than they otherwise would via information on the food packaging. The general research methodology is clearly described (the photos really helped) and appropriate to achieve the study aims. The paper is interesting to read and well-written. I have some suggestions to improve the rigor of the analysis and the clarity of the paper.

1) My biggest concern with the manuscript is that the authors have not provided analysis results and/ summary tables from their qualitative analysis of the focus group interviews.

a) For example, I was expecting there to be a table with the major themes that emerged, and the number of participants or comments that supported that theme. Instead, the authors just state their conclusions from the focus groups without providing the reader any evidence that their conclusions are valid.

*Response: We thank the reviewer for the comment. In the revised version we have included the coding guide (S2 File) as well as the results of the double-entry matrix coding (S4 File).*

d) On line 225, you state that "results on analytics codes are presented for each of the FOPL tested." Where are these results presented? Do you mean the descriptions that follow later in the main body of the paper? I would expect these results to include quantitive statements, something like: 18 out of the 20 people who mentioned the Mexican GDA stated that they did not find it helpful. Instead the authors write (on lines 251-253, "Food purchasing decisions were generally based on price... whereas GDA was generally not mentioned as a purchasing criteria." And later on in that paragraph, "Older adults also tended to report using GDA more frequently for health reasons." It is those types of statements that could be more rigorous by providing specific counts and agreement percentages. I imagine this information be contained in a table. This comment applies to all of the food labels that are discussed (Healthy Choices, 5-CNL, MTL, HSR, WL.

*Response: We thank the reviewer for the comment. As mentioned above, we have now included a the results of the double-entry matrix coding, which contain the quotes for each analysis category per FOPL (S4 File). We have also clarified the statement in line 225 to indicate that this information is included in the supplementary materials and the interpretation are included in the main text in a narrative form.*

*However, we respectfully disagree with the revierwers’ suggestion to include quantitative statements becuase:*

1. *The focus group was not a group interview where all participants were asked to respond to a particular question (this often happens in marketing studies). Qualitative methodologies are descriptive, exploratory, and oriented towards exposing meanings from participants’ perspectives (Cortes-Caseres, 2008). Focus Group is a type of in-depth interview accomplished in a group, whose meetings present characteristics defined with respect to the proposal, size, composition, and interview procedures. The focus or object of analysis is the interaction inside the group. Thus, the focus group dynamic engaged a group of participants in a conversation. Mentioning the number of who said X or Y would not represent any sense of agreement or prevalence.*
2. *In line with the former, our sample was not meant to provide representativeness among a specific group of people. The dynamic was designed to capture the expressions of the different groups for medium and low socioeconomic level. Likewise, the quantification of what each participant says would not represent the population studied. People who refer a certain testimony within a focus group represent that specific profile of the focus group in which they are participating, regardless the actual representativeness of this particular profile among the population. Therefore it is not feasible to conduct a survey to know how many of the group agree with a certain testimony of other participants.*
3. *Based on editors’ comment, results are presented following the Consolidated Criteria for Reporting Qualitative Reserarch (COREQ), which does not consider quantifying statements*

*We have included additional information on the limitations of focus groups in the discussion section to address the former comments (Line 529).*

*Reference:*

*Cortés, F.; Escobar, A.; Gonzáles de la rocha, M. Método Científico y política social: a propósito de lss evaluaciones cualitativas de los programas sociales; AC., E.C. de M., Ed.; 1st ed.; México, D.F., 2008; ISBN 968-12-1329-7.*

b) The authors could provide the list of questions asked during the focus groups (perhaps as an appendix), and their data collection instrument that was mentioned on line 119.

*Response: Thanks, we have now included the semi-structured topics guide that the moderator used during focus groups as a supplementary file (S2 File). Reference to the guide is made in Line 193. We have also included a couple of detonating questions in the text (Line 198).*

c) On line 224, you state that the researchers reached similar conclusions. Do you have statistical analysis or some kind of way of quantifying this claim, such as kappa scores indicating agreement?

*Response: We thank the reviewer for the suggestion. Although in qualitative methodologies consensus can be verified by using statistical tests, most of the techniques used to reach consensus between encoders considers other non-quantitative alternatives (Morse, 2015). For our analysis we used a codebook wich is a tool to prevent codes from seeming abstract or vague. As mentioned above, this is included in S2 File. Also we used the peer review technique, which consists on multiple meetings between researchers to discuss results and their interpretation, until consensus is reached (Morse, 2015; Liamputtong, 2016). We have now included additional information of this process (Lines 238).*

*References*

*Morse JM. Critical Analysis of Strategies for Determining Rigor in Qualitative Inquiry. Qual Health Res. 2015;25:1212–22.*

*Liamputtong P. Qualitative Research methods. 4th edition. Ausrtralia; 2016.*

2) I was not familiar with some of the terms used in the manuscript. The authors could consider using different terms or defining them.
a) Free sugars (as opposed to just "sugars")

b) Acceptability. What does this mean with regard to food labels? Please define or use a different word that is more obvious in its meaning.

*Response: In the revised version we replaced the term of “free sugars” by the term “sugar” throught the text and we included a definition for the term “acceptability” (line 208).*

What is a "Gessell chamber"? Why does it matter for your study? If it is important, please describe what it is and why it helps your research to have had the focus group sessions take place in Gessell chambers.

*Response: In the revised version we included a description of the relevance of the Gessell chamber (line 156).*

3) The paper would benefit from a careful copy-editing.

a) There are a few places that seem to be missing commas (introductory phrases such as "In Mexico,.." on page 3 introduction, line 60 and again on page 4, line 78. In June 2016, On page 4, line 71.

*Response: Corrected*

b) There are a few places where the grammar is slightly off.
   i) Line 460 "participants considered this label did not provide..." I think the sentence would read better if you either inserted the word "that" after considered, or better yet, rewrote it to be: "participants felt that this label did not provide.."

*Response: Corrected*

ii) Line 462 "in consequence" should be "consequently"

*Response: Corrected*

iii) Line 504 "which has been proved to overestimate" should be "which has been shown to.."

*Response: Corrected*

iv) Line 506 "characteristics resemble to those of Mexican..." Delete "to" in that phrase.

*Response: Corrected*

v) Line 508 "have a limited time to purchase". I suggest deleting "a".
*Response: Corrected*

4) In future research, you mention testing the labels with actually food purchases. There is prior research that does investigate actual food purchases as impacted by labels, although perhaps not in Latin America. For example, Mazza, M. C., Dynan, L., Siegel, R. M., & Tucker, A. L. (2018). Nudging Healthier Choices in a Hospital Cafeteria: Results From a Field Study. Health Promotion Practice, 19(6), 925–934. [https://doi.org/10.1177/1524839917740119](https://eur01.safelinks.protection.outlook.com/?url=https%3A%2F%2Fdoi.org%2F10.1177%2F1524839917740119&data=02%7C01%7C%7Cb23dcc7ca1344026be2f08d72c9948b0%7C84df9e7fe9f640afb435aaaaaaaaaaaa%7C1%7C0%7C637026909173982550&sdata=%2Fg87oS5ujWvg9j54X3H7ZRfMVLHJ2uTye0BfAabenuY%3D&reserved=0). investigates the use of traffic light on food purchases in a workplace cafeteria.

*Response: We agree with the reviewer and are grateful for the references, however we meant this sort of research in Latin Amerca. We have referenced this study in the conclusion and highlighted that this sort of research should be conducted in the Latin American context (Line 549)*

I wish the authors the best of luck in revising their manuscript.

*Thanks*
